# Integrative network pharmacology, transcriptomics, and proteomics reveal the material basis and mechanism of the Shen Qing Weichang Formula against gastric cancer

**DOI:** 10.1186/s13020-025-01091-4

**Published:** 2025-03-29

**Authors:** Yi Wang, Xiaoyu Sun, Mingming Ren, Fangqi Ma, Ruohan Zhao, Xiaohong Zhu, Yan Xu, Nida Cao, Yuanyuan Chen, Yongfu Pan, Aiguang Zhao

**Affiliations:** 1 Department of Oncology, Longhua Hospital, Shanghai University of Traditional Chinese Medicine, South Wanping Rd. 725, Shanghai, 200032 China; 2 Cancer Institute of Traditional Chinese Medicine, Longhua Hospital, Shanghai University of Traditional Chinese Medicine, South Wanping Rd. 725, Shanghai, 200032 China

**Keywords:** Shen Qing Weichang formula, Gastric cancer, Network pharmacology, Transcriptomics, Proteomics, PI3K-AKT signaling pathway, MAPK signaling pathway, Apoptosis, Paclitaxel

## Abstract

**Background:**

Gastric cancer (GC) is a common malignancy with poor prognosis and lack of efficient therapeutic methods. Shen Qing Weichang Formula (SQWCF) is a patented traditional herbal prescription for GC, but its efficacy and underlying mechanism remains to be clarified.

**Purpose:**

To explore the efficacy and potential mechanism of SQWCF in treating GC.

**Methods:**

A subcutaneous transplantation tumor model of human GC was established for assessing SQWCF’s efficacy and safety. A comprehensive strategy integrating mass spectrometry, network pharmacology, omics analysis, and bioinformatic methods was adopted to explore the core components, key targets, and potential mechanism of SQWCF in treating GC. Molecular docking, immunohistochemistry, quantitative real-time PCR, and western blot were applied to validation.

**Results:**

In the mouse model of GC, SQWCF effectively suppressed the GC growth without evident toxicity and enhanced the therapeutic efficacy of paclitaxel. Network pharmacology and molecular docking based on mass spectrometry showed that key targets (CASP3, TP53, Bcl-2, and AKT1) and core active components (Calycosin, Glycitein, Liquiritigenin, Hesperetin, and Eriodictyol) involved in the anti-GC effect of SQWCF had stable binding affinity, of which AKT1 ranked the top in the affinity. Validation based on network pharmacology and omics analysis confirmed that PI3K-AKT and MAPK signaling pathways, as well as downstream apoptosis pathway, explained the therapeutic effects of SQWCF on GC. In addition, family with sequence similarity 81 member A (FAM81A) was identified as a novel biomarker of GC that was aberrantly highly expressed in GC and associated with poor prognosis by bioinformatic analysis, and was an effector target of SQWCF at both mRNA and protein levels.

**Conclusion:**

This study uncovers a synergistic multi-component, multi-target, and multi-pathway regulatory mechanism of SQWCF in treating GC comprehensively, emphasizing its potential for therapeutic use and providing new insights into GC treatment.

**Graphical abstract:**

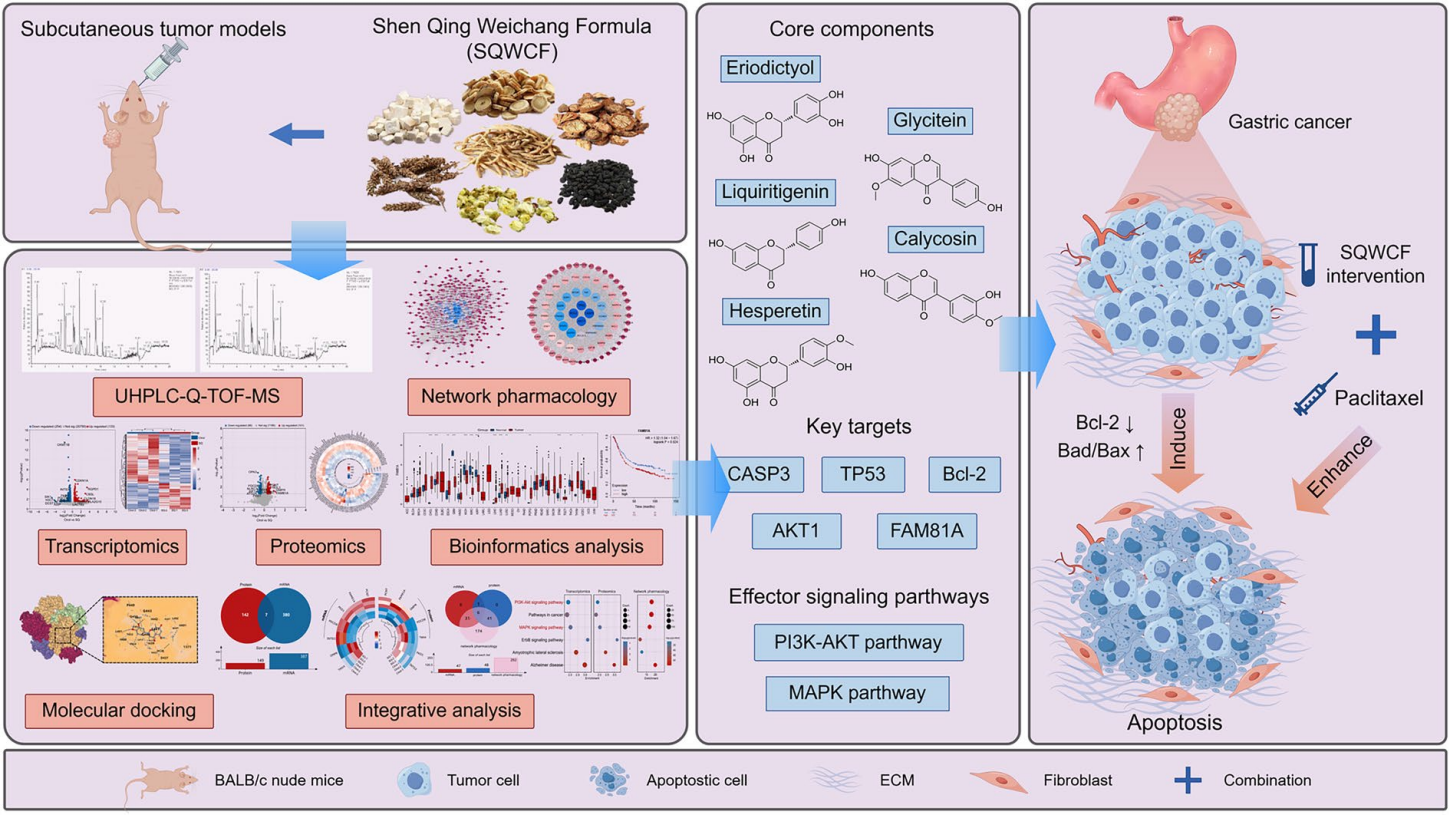

**Supplementary Information:**

The online version contains supplementary material available at 10.1186/s13020-025-01091-4.

## Introduction

Gastric cancer (GC) is one of the deadliest malignant tumors, ranking fourth in global cancer-related deaths, with nearly 800,000 deaths a year [1]. Common treatments for GC mainly include surgery, chemotherapy, targeted therapy, immunotherapy, etc. [2]. However, without distinguishing clinical indications available at present, about 75% of patients were diagnosed at advanced stage [3], making surgery unavailable. Due to extremely heterogeneous etiology and pathogenesis, there are limited beneficiaries from chemotherapy, targeted therapy, and immunotherapy represented by immune checkpoint inhibitors (ICIs) [4, 5]. Moreover, GC patients are unable to benefit from these therapies in a long term owing to the existence of drug resistance [6], highlighting a necessity for exploring new therapeutic agents for GC.

Traditional Chinese medicine (TCM) exhibits various advantages such as multi-component, multi-target approaches and excellent safety profiles, gaining extensive attention worldwide [7, 8] and offering new therapeutic options for GC [9]. One such formulation for GC treatment is Shen Qing Weichang Formula (SQWCF; patent No.: 202110353781.2), which consists of *Pseudostellariae Radix* (Taizishen), *Poria Cocos (Schw.) Wolf* (Fuling), *Arum Ternatum Thunb* (Banxia), *Sargentodoxae Caulis* (Daxueteng), *Prunellae Spica* (Xiakucao), *Mume Flos* (Baimeihua), and *Fructus Ligustri Lucidi* (Nvzhenzi). SQWCF has a great developing prospect for GC treatment by regulating Qi, strengthening spleen, and removing toxic substances to dissipate mass. However, the efficacy and underlying mechanism of SQWCF in GC remain unclear.

The serine/threonine kinase (AKT), a proto-oncogene, plays a vital regulatory role in multiple cellular functions [10, 11], which is activated by stimulating phospha-tidylinositol (3,4,5) trisphosphates (PIP3) induced by phosphoinositide 3-kinase (PI3K) [12]. Over-activation of PI3K-AKT signaling pathway can regulate apoptosis by inhibiting pro-apoptotic protein Bcl2-associated agonist of cell death (Bad) [13], resulting in tumor cell survival and drug resistance [14, 15]. Therefore, targeting PI3K-AKT pathway has gradually become a promising anticancer strategy, including GC [16, 17].

The mitogen-activated protein kinase (MAPK) family, as one of the important transmitters of cell information [18], consists of three subfamilies: extracellular regulated protein kinase (ERK), c-Jun N-terminal kinase (JNK), and p38 MAPK [19]. Specifically, ERK signaling pathway regulates cell growth and differentiation [20], while JNK and p38 MAPK signaling pathways are involved in modulating stress responses such as inflammation and apoptosis [21], all of which are essential in the process of malignancies. Abnormal activation of critical proteins in MAPK pathway may be a determinant of GC [22], suggesting that effective suppression of this pathway can offer a profound anti-tumor strategy for GC [23].

In this study, we evaluated the anticancer efficacy and safety of SQWCF in an established mouse model of GC and revealed the underlying core active compounds, key gene targets, and molecular mechanism through comprehensively designed exploratory and validation experiments. Our results demonstrated that SQWCF exhibited significant inhibitory role without evident toxicity and a synergistic effect with paclitaxel (PTX) in vivo. Mechanistically, PI3K-AKT and MAPK pathways, as well as the downstream apoptosis pathway, play a critical role in the anti-GC activity of SQWCF. Moreover, the family with sequence similarity 81 member A (FAM81A) would be predicted to be a potential biological marker of GC, which is aberrantly highly expressed and associated with poor prognosis in GC, presenting as an effector target for SQWCF. We believe that this study will decipher the potential of SQWCF for GC and provide valuable clues for expanded application of TCM in GC treatment.

## Materials and methods

### Chemicals and reagents

5-Fluorouracil (5-FU) was bought from MCE (MedChemExpress, Shanghai, China). Paclitaxel (Albumin Bound) was purchased from Jiangsu Hengrui Pharmaceuticals Co., Ltd. (Jiangsu, China). Antibodies Bcl-2, Bax, Bad, caspase3, cleaved-caspase3, cleaved-PARP, PARP, p-ERK, ERK, p-AKT, AKT, p-PI3K, PI3K, JNK, p-JNK and GAPDH were purchased from Cell Signaling Technology (Boston, USA). Antibodies p-p38 MAPK and p38 MAPK were purchased from Proteintech (Wuhan, China). Antibody β-actin was from HuaBio (Hangzhou, China). FAM81A was bought from GeneTex (Alton, USA). All other reagents and chemicals used in the present study were obtained from local suppliers.

### Herbal decoction and quality control research

The herbal decoction SQWCF consists of seven herbs listed in Table 1. All herbs were supplied by the Longhua Hospital Shanghai University of TCM (Shanghai, China). Initially, all the herbs were soaked in water for 30 min (1:10, w/v) to obtain the medicinal juice after two times of decoction. Subsequently, the two extracts were mixed, concentrated, dried and sieved to obtain a powdered form suitable for storage. High-performance liquid chromatography (HPLC) was used to determine the fingerprints of 9 batches of formula granules of SQWCF. Detailed methods and results are presented in Supplementary materials (Additional file 1: Fig. S1 and Additional file 4: Table S1).

**Table 1:** Composition of SQWCF decoction

Latin name	English name	Local name	Used part	Weight (g)
*Pseudostellariae Radix*	Heterophylly Falsestarwort Root	Taizishen	Root	12 g
*Poria Cocos (Schw.) Wolf*	Indian Bread	Fuling	Sclerotium	30 g
*Arum Ternatum Thunb*	Pinellia Tuber	Banxia	Root and rhizome	9 g
*Sargentodoxae Caulis*	Sargentgloryvine Stem	Daxueteng	Vine and stem	30 g
*Prunellae Spica*	Common Selfheal Fruit-Spike	Xiakucao	Flower	9 g
*Mume Flos*	Plum Flower	Baimeihua	Flower	9 g
*Fructus Ligustri Lucidi*	Glossy Privet Fruit	Nvzhenzi	Fruit	9 g

### Cell lines and cell culture

The human GC cell line MKN45 was purchased from the Cell Bank of the Chinese Academic of Science (Shanghai, China). MKN45 cells were cultured in RPMI-1640 medium (BasalMedia, Shanghai, China), containing 10% fetal bovine serum (FBS, ExCell Bio, China) and 1% penicillin–streptomycin solution (Gibco, USA), at 37 ℃ in a humidified atmosphere with 5% CO_2_. The medium was changed every 2 days, and the third to eighth generation of cells were used for the experiments.

### Subcutaneous transplantation tumor model of human GC and experimental design

Male BALB/c nude mice of 4 weeks old (16–18 g) were provided by Shanghai Laboratory Animal Center (Shanghai, China). The animals were kept in standard cages at 20–25 °C and 40–60% of humidity under a 12/12 h light/dark cycle for one week of adaptive feeding. All animal experiments were performed according to the protocol of the Experimental Animal Ethics Committee of Shanghai University of TCM (Approval No.: PZSHUTCM2304100001). An appropriate number of animals were used to establish a subcutaneous transplantation tumor model. MKN45 cells (2*10^6^) were suspended in 100 μL phosphate buffered saline and subcutaneously implanted into the right armpit of mice. Mice were randomly grouped according to the experimental requirements 4 days after injection.

For pharmacodynamic evaluation of SQWCF, mice were randomly divided into 5 groups: control group (normal saline, 0.2 ml/20 g), SQWCF high-dose group (SQ-H, 476 mg/kg), SQWCF medium-dose group (SQ-M, 238 mg/kg), SQWCF low-dose group (SQ-L, 119 mg/kg), with all interventions given by oral gavage (once a day); as well as 5-fluorouracil group (5-FU, 25 mg/kg, 3 times a week, intraperitoneal injection) [24], with 6 mice per group. Among them, 5-FU served as a positive control against GC. The dose used for the SQ-M group was converted from clinical doses using the body surface area normalization method [25], which was equivalent to 108 g of herbs for an adult patient with GC per day in a clinical setting. Besides, the high and low groups were 2 times and 0.5 times that of the SQ-M group, respectively.

For pharmacodynamic evaluation of SQWCF combined with PTX, mice were randomly divided into the control group (normal saline, 0.2 ml/20 g, once a day, oral gavage), SQWCF group (SQ, 238 mg/kg, once a day, oral gavage), PTX group (PTX, 5 mg/kg, 2 times a week, intraperitoneal injection) [26, 27] and PTX + SQWCF (PTX, 5 mg/kg, 2 times a week, intraperitoneal injection; SQ, 238 mg/kg, once a day, oral gavage), with 6 mice per group. The tumor size and body weight of mice were recorded every three days. Tumor volume was estimated by length × width^2^ × 0.5.

After the termination of experiments, tumor and organ tissues as well as blood samples were harvested from mice euthanized by cervical dislocation under anesthesia using pentobarbital sodium. Tumors were collected, weighed, and photographed, then fixed in 4% paraformaldehyde along with liver and kidney tissues, followed by hematoxylin and eosin (H&E) staining and TdT-mediated dUTP Nick-End Labeling (TUNEL) staining. Detailed methods are described in the Supplementary material. Serum samples were drawn for assessing the biochemical function of the liver and kidney.

### Immunohistochemistry analysis

The tumor tissues were taken for dewaxing and hydration, and the non-specific protein was blocked by 5% goat serum. Paraffin sections were incubated with primary antibodies and secondary antibodies, then developed with 3.3’-diaminobenzidine, and finally counterstained with hematoxylin. After dehydration and fixation, images were captured under light microscopy (Nikon, Japan).

### Analysis of SQWCF chemical composition

The chemical compounds of SQWCF were identified by ultra-high performance liquid chromatography-quadrupole time-of-flight mass spectrometry (UHPLC-Q-TOF-MS). Briefly, 2 mg of sample was dissolved in 1 mL of methanol, followed by ultrasound for 20 min and centrifugation to obtain the supernatant. Then, 0.5 mL of the supernatant was taken and mixed with 1.5 mL of water for analysis on UltiMate 3000 UHPLC with a C18 column (1.9 μm, 2.1 mm*100 mm, Waters, USA) at 35 ℃. The drug analysis was performed using a flow rate of 0.3 ml/min and an injection volume of 10 μL. The 0.1% formic acid/acetonitrile (B)-0.1% formic acid/water (A) were used for gradient elution with the program as follows: 5%-100% B (0–5 min); 100%-100% B (5–17 min); 100%-5% B (17–20 min). The detection wavelength was 254 nm. Then, the mzCloud database (https://www.mzcloud.org/) was searched to obtain the component list for the screening of components with a high matching degree and peak area exceeding 1*10^7^.

### Prediction and screening of targets

The active components identified by UHPLC-Q-TOF–MS were imported into the Traditional Chinese Medicine Systems Pharmacology (TCMSP) (https://old.tcmsp-e.com/tcmsp.php), and screened according to oral bioavailability (OB) value ≥ 30% and drug-like properties (DL) value ≥ 0.18 [28]. The potential targets of the screened active components were predicted through the SwissTargetPrediction (http://www.swisstargetprediction.ch/). Targets for GC were collated from the GeneCards (https://www.genecards.org/), OMIM (https://www.omim.org/), and DisGENET (https://www.disgenet.org/), using “gastric cancer” as the search term. All targets were corrected by the Uniprot (https://www.uniprot.org/) database, and the intersection targets between SQWCF and GC were determined by the Venn diagram, which was selected as the potential candidate targets for SQWCF against GC.

### Study on protein–protein interaction (PPI) network

To further explore the interactions among the common targets, we utilized the STRING (https://string-db.org/) for analysis (minimum interaction score > 0.4) with the sample type specified as "Homo sapiens". The results were imported into Cytoscape (v 3.7.1) and visualized. The plugin CytoNCA was employed to calculate "Betweenness Centrality" (BC), "Degree Centrality" (DC), "Closeness Centrality" (CC), and "Eigenvector Centrality" (EC) for identifying core components and key targets with high connectivity in the PPI network.

### Gene ontology (GO) and Kyoto encyclopedia of genes and genomes (KEGG) analysis

GO analysis and KEGG pathway analysis were constructed using the Metascape database (http://metascape.org/). A significance level of *p* < 0.05 was employed as the critical threshold for analysis.

### Transcriptomics study

The collected tumor tissues from Control (n = 3) and SQ-M (n = 3) groups were used to transcriptomics analysis. After total RNA extraction using Trizol (Thermo Fisher, MA, USA), the integrity and potential contamination of RNA were assessed using Bioanalyzer 2100 (Agilent, CA, USA). NanoDrop 2000 Spectrophotometer (NanoDrop, DE, USA) was utilized to determine purity and concentration. Then, RNA libraries were sequenced on the Illumina NovaSeq™ 6000 platform by TIANGEN (Beijing, China). Details are available in Supplementary materials. Genes with Fold Change (FC) > 1.2 and *p* value < 0.05 by DESeq2 (v 1.4.5) were designated as differentially expressed genes [29].

### Gene set enrichment analysis (GSEA)

The gene set derived from the transcriptomics study and KEGG gene sets were uploaded to GSEA 4.2.3 software to get the associated pathway terms. The gene sets from GSEA were excluded when their sizes were less than 5 or larger than 2000. Normalized enrichment score (NES) > 1 and false discovery rate (FDR) < 0.25 were applied to evaluate enrichment magnitude and statistical significance, respectively.

### Proteomics study

The tumor tissues from the control (n = 3) and SQ-M (n = 3) groups were subjected to proteomic analysis. The collected tissues were incubated in lysis buffer, sonicated, and centrifuged. After that, the protein samples were reacted with triethylammonium bicarbonate buffer, tris (2-carboxyethyl) phosphine, iodoacetamide, and pre-cooled acetone successively, followed by centrifugation to obtain precipitation and digestion overnight with trypsin. Subsequently, the peptide was desalted, quantified, and labeled with tandem mass tags (TMT) reagent (Thermofisher, Waltham). Labeled peptides were analyzed by online nano-flow liquid chromatography tandem mass spectrometry performed on an Evosep One system (Evosep, Denmark). The spectral data were analyzed by ProteomeDiscoverer™ Software 3.0 and compared with the UniProt protein database. Proteins were quantified using MaxQuant software. Quantitative proteomics mass spectrometry was performed by TIANGEN (Beijing, China). Details are available in Supplementary materials. Quantitative protein results were statistically analyzed by Student *t*-test. FC > 1.2 and *p* value < 0.05 were employed to determine the statistical significance of protein expression differences [29].

### FAM81A expression analysis

The Cancer Genome Atlas (TCGA) and Genotype-Tissue Expression (GTEx) databases were searched for screening and pooling FAM81A mRNA data across 33 cancer types and corresponding paracancer, as well as normal samples. The obtained data were analyzed statistically using R software (v 3.6.3) was used to perform statistical analysis, and the results were visualized by “ggplot2” (v 3.3.3) package. Abbreviations for all cancers analyzed was listed in Additional file 4: Table S4. The Wilcoxon rank-sum test was used for inter-group comparison, and *p* < 0.05 was considered statistically significant.

### Prognostic assessment of gene expression

The prognostic significance of gene expression in GC was assessed by Kaplan–Meier Plotter (http://kmplot.com/analysis/), a database that integrates data from Gene Expression Omnibus (GEO), European Genome-Phenome Archive (EGA), TCGA and other databases to discover and verify survival biomarkers [30]. The dichotomization of gene expression levels was performed by selecting the “auto-selection of the best cut-off” option and the hazard ratio with 95% confidence intervals and log-rank *p* value are calculated. And *p* < 0.05 was considered statistically significant.

### Quantitative real-time PCR (Q-PCR) analysis

Total RNA was isolated using the Ultrapure RNA Kit (TIANGEN Biotech China) following the manufacturer's protocol. The PrimerScript reverse transcription reagent kit (Vazyme Biotech, Nanjing, China) was used to reverse total RNA to cDNA. Reactions were performed using the Power SYBR Green PCR MasterMix (Vazyme Biotech, Nanjing, China) on the Quant Studio 5 thermocycler (Applied Biosystems, USA) following the instrument instructions. β-actin mRNA served as a control. The primer sequences are listed below: FAM81A, 5′-CTTAGCCAGGCTGTTCTTGG − 3′, and 5′-CCAGCGTCTTTAAGGCAGAA − 3′; β-actin, 5′-ACTCTTCCAGCCTTCCTTCC-3′, and 5′-CAATGCCAGGGTACATGGTG-3′.

### Molecular docking

The 3D structure of target proteins (TP53, CASP3, Bcl-2, and AKT1) was downloaded from the Protein Data Bank (PDB) website (https://www.rcsb.org/), and FAM81A was obtained from AlphaFold Protein Structure Database (https://alphafold.com/). The PyMOL (v 2.5.0) was applied to remove ligands and water molecules. The structure of active components was obtained from the TCMSP platform. PDB files of components and active site of proteins were converted into “pdbqt” format using AutoDockTools (v1.5.6). At last, Autodock Vina (v 1.1.2) was used for molecular docking, and the results were visualized using PyMOL (v 2.5.0).

### Western blot

The total protein was extracted and quantified by a BCA kit (Epizyme, Shanghai, China). Equal amounts of proteins were resolved on SDS-PAGE gel and then transferred to the PVDF membrane (Merck Millipore Ltd., Tullagreen, Ireland). Subsequently, PVDF membranes were incubated with primary antibodies and conjugated with secondary antibodies. Signals were visualized by ECL reagent (ShareBio, Shanghai, China) and photographed by Tanon 5200 visualizer (Tanon, Shanghai, China).

### Statistical analysis

The results were expressed as mean ± standard deviation (SD) and analyzed by one-way analysis of variance (ANOVA) and the student *t*-test in GraphPad Prism version 8.0 (GraphPad Software, California, USA). A *p* value < 0.05 was considered statistically significant.

## Results

### SQWCF suppressed GC growth with notable safety in vivo

**Fig. 1:**
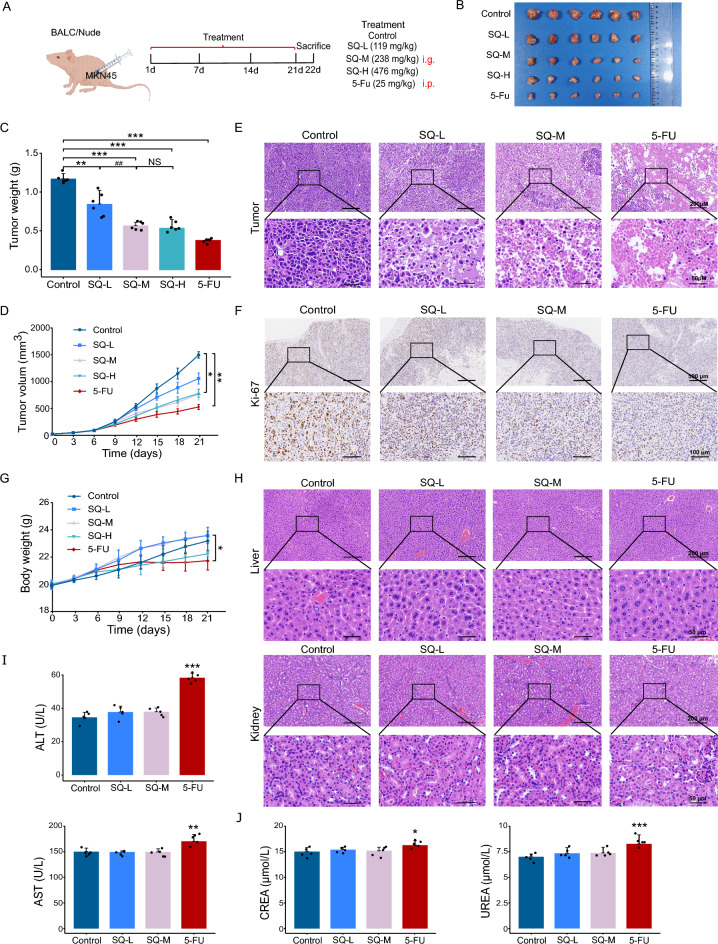
SQWCF suppressed the growth of GC in vivo. **A** Experience design. **B** The physical appearance and size of the tumor tissues. **C** The tumor weight statistics. **D** The tumor volume statistics. **E** H&E staining of the tumor tissues. **F** IHC staining of Ki-67 in tumor tissues. **G** The body weight statistics. **F** HE staining of liver and kidney tissues. **I–J** Levels of liver and renal function indicators in serum. Data were presented as mean ± SD. Compared to control: *p < 0.05, **p < 0.01, ***p < 0.001. Compared to SQ-M: ^##^*p* < 0.01, NS, no significant

To evaluate the anticancer effect and safety of SQWCF in GC, a subcutaneous tumor model in BALB/c nude mice was constructed, with five treatment regimens administered (Fig. 1A). In Fig. 1B–D, there was a significant decrease in tumor volume and weight in the 5-FU and SQWCF groups. Within three dosages, SQWCF suppressed tumor growth; however, the anti-GC effect at the low dosage was not as obvious as other dosages. There was no obvious difference in the efficacy between the medium and high dosages. Accordingly, 238 mg/kg was identified as the optimal therapeutic dosage of SQWCF in treating GC and used for subsequent experiments. H&E and Kiel 67 (Ki-67), a marker of proliferation, staining showed that SQWCF or 5-FU treatment significantly inhibited the proliferation of GC cells (Fig. 1E–F). Moreover, in Fig. 1G–J, there were no differences in body weight or the histological morphology and biochemical function of liver and kidney after SQWCF treatment. 5-FU-treated mice showed notable weight reductions along with a significant rise in liver and renal function indicators, suggesting that the use of SQWCF was safe for treating GC at the animal experimental level. Altogether, SQWCF showed a pronounced inhibitory effect on the growth of GC, with no apparent signs of toxicity in vivo.

### Identification of SQWCF chemical composition

**Fig. 2:**
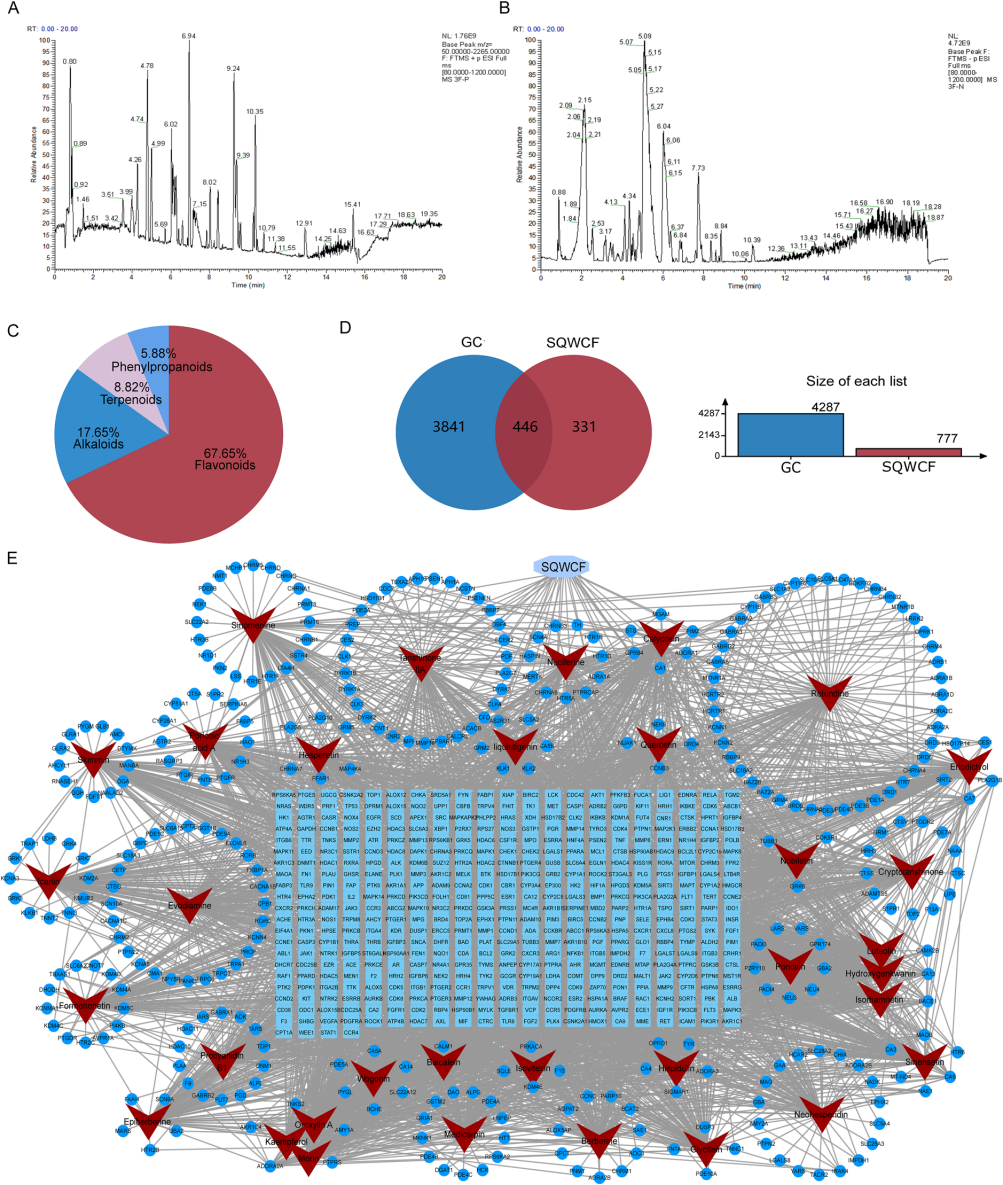
The SQWCF-target network analysis based on identification of chemical components. **A** The chromatogram of SQWCF in positive mode. **B** The chromatogram of SQWCF in negative mode. **C** Classification and quantity of chemical components with OB value ≥ 30% and DL value ≥ 0.18. **D** Intersections of SQWCF and GC targets. **E** The compounds-targets network of SQWCF against GC. Red represents chemical compounds of SQWCF, and blue represents their related targets. The shallow blue represents SQWCF-GC common targets, the dark blue represents unique targets. Edges between nodes represent target-target associations in the network

The base peak chromatogram obtained by UHPLC-Q-TOF–MS analysis in both positive and negative ion modes was shown in Fig. 2A–B. A total of 232 compositions were preliminarily caught and shown in Additional file 4: Table S2. According to the TCMSP database, 34 chemical components with OB value ≥ 30% and DL value ≥ 0.18 were screened for network pharmacological analysis (Additional file 4: Table. S3), including 23 flavonoids, 6 alkaloids, 3 terpenoids, and 2 phenylpropanoids (Fig. 2C).

### Analysis of the mechanism of SQWCF against GC by network pharmacology

A total of 4,287 different genes associated with GC were obtained from GeneCards, OMIM, and DisGeNET databases, and 777 component-related targets were identified from the SwissTargetPrediction database. In Fig. 2D, intersections between the two datasets identified 446 common targets for SQWCF in the treatment of GC.

To gain a deeper understanding of the complicated relationship between compounds and targets, a compound-target network was established using Cytoscape 3.7.1. In Fig. 2E, the network consisted of 812 nodes and 3,556 edges. Network topology analysis identified five core compounds of SQWCF against GC, including Calycosin, Glycitein, Liquiritigenin, Hesperetin, and Eriodictyol. The topological properties of the five compounds are shown in Table 2.

**Table 2:** The topological analysis of SQWCF in the compound-target network

Compound	Degree Centrality	Betweenness Centrality	Closeness Centrality	Eigenvector Centrality
Hesperetin	105	20,325.918	0.37851518	0.17429148
Calycosin	104	6258.504	0.37894145	0.119466595
Glycitein	104	24,016.336	0.37851518	0.108245894
Liquiritigenin	102	21,135.543	0.37766555	0.10385731
Eriodictyol	102	15,992.193	0.37766555	0.1168284

**Fig. 3:**
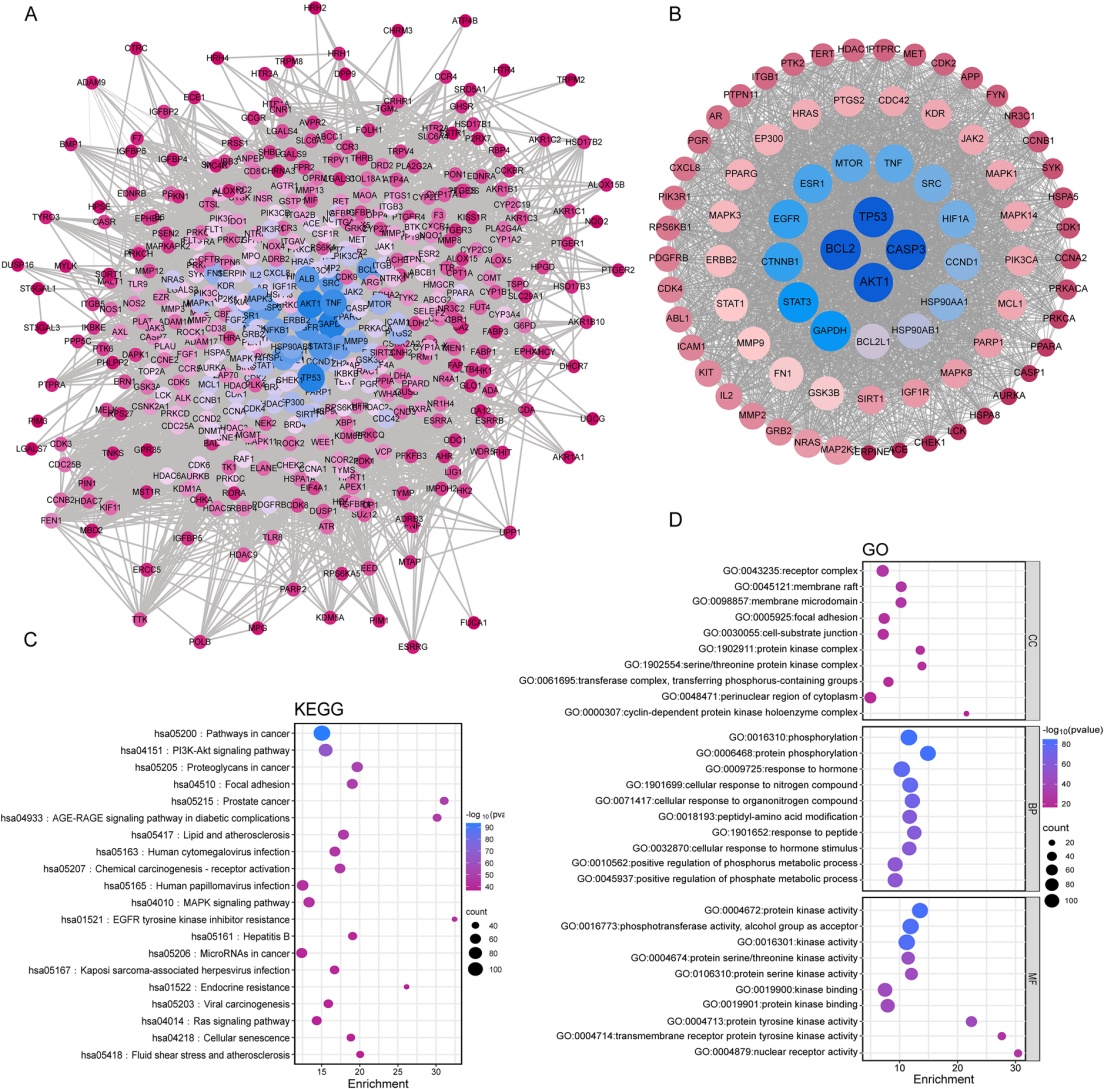
The identification of SQWCF’s key targets and KEGG and GO enrichment analysis.** A** Interaction network of common gene targets. **B** Visualization network of key targets. The color intensity and the diameter of each circle reflects the degree of criticality of each gene target, with darker colors and larger circular areas indicating greater criticality. Edges between nodes represent target-target associations in the network. **C–D** KEGG and GO enrichment analysis of SQWCF-GC common target genes

Then, a PPI network was established using the 446 common targets of SQWCF and GC, as depicted in Fig. 3A, containing 446 nodes and 11,635 edges. Subsequently, the key targets (AKT1, BCL2, TP53, and CASP3) of SQWCF against GC were identified according to the degree value (Fig. 3B).

In Fig. 3C–D, GO and KEGG enrichment analyses were performed for further clarification of the biological functions and potential mechanism of the 446 common targets. KEGG pathway enrichment showed that common target genes were primarily involved in pathways in cancer, the PI3K-AKT signaling pathway, proteoglycans in cancer, and focal adhesion. GO analysis indicated significant enrichment in receptor complex, membrane raft, phosphorylation, protein phosphorylation, protein kinase activity, phosphotransferase activity, and alcohol group as acceptor. Collectively, the network pharmacology analysis predicted core active components and key targets involved in ‘the anti-GC effect of SQWCF, providing a valuable direction for further exploration of the underlying mechanism.

### Molecular docking of the interaction between compounds and targets of SQWCF

**Fig. 4:**
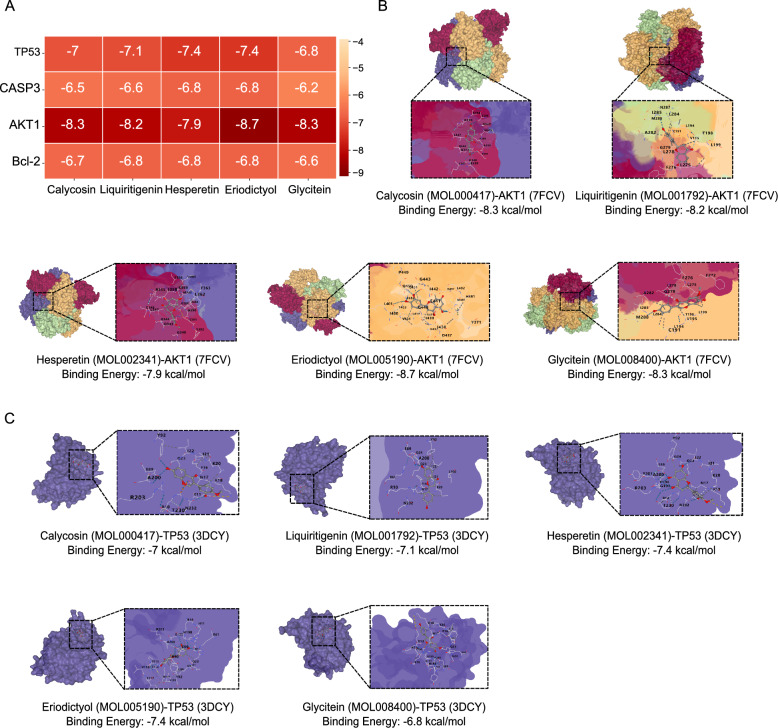
Molecular docking results. **A** Heatmap of molecular docking scores. **B**–**C** The docking of the two targets with the best docking activities (AKT1 and TP53) and compounds (Calycosin, Glycitein, Liquiritigenin, Hesperetin, and Eriodictyol). All pictures show the 3D docking of ligands in the active binding pocket (left or up), with the hydrophobic effect area and the 2D interaction patterns between the ligands and proteins (right or down)

Molecular docking can predict receptor-ligand binding patterns and affinity based on structure, with more stable interaction confirmed when there is a lower docking value [31]. Therefore, a molecular docking analysis was employed to evaluate the capacity of binding between the components and targets of SQWCF. The docking models and scores are shown in Fig. 4 and Additional file 2: Fig. S2. In detail, the docking scores of the key targets and the core compounds of SQWCF were all below − 5 kcal/mol, indicating stable target-compound binding capacity. Among them, AKT1 and TP53 showed the optimal docking activities to the five core compounds of SQWCF (Fig. 4B–C).

### Identification of genes and pathways related to the effect of SQWCF against GC via transcriptomics analysis

**Fig. 5:**
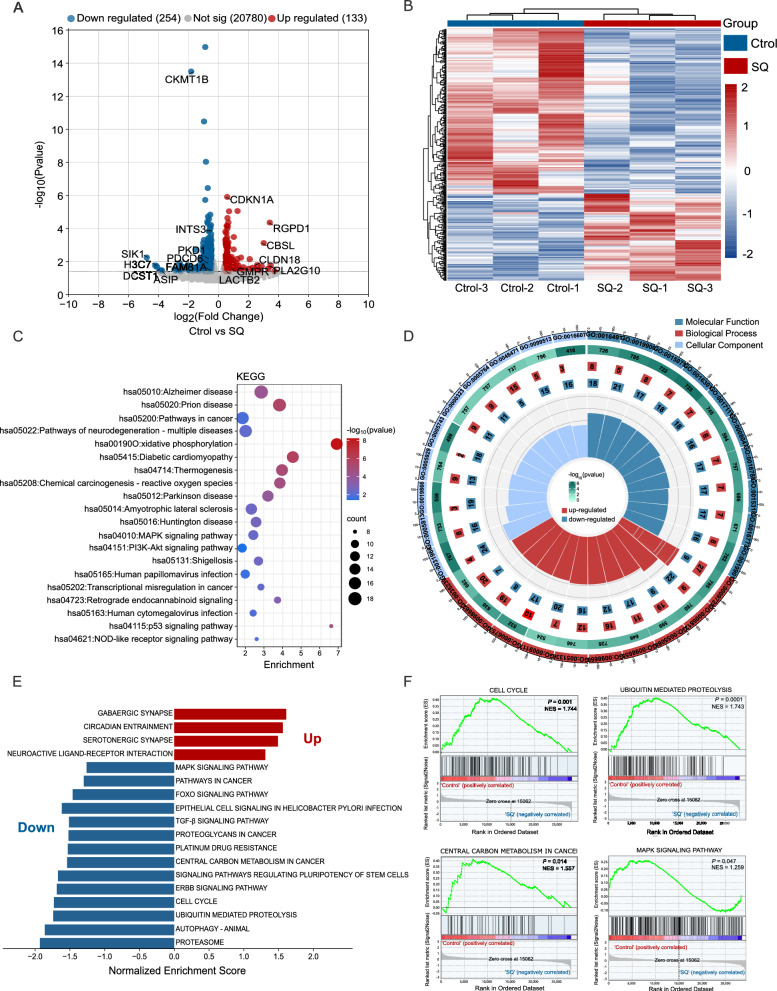
Transcriptomics analysis for the mechanisms of SQWCF against GC.** A** Volcano plot of DEGs between control and SQWCF group. **B** Heatmap of the different abundance genes among control and SQWCF group. **C–D** KEGG and GO enrichment analysis of DEGs.** E–F** GSEA analysis of DEGs. NES, normalized enrichment score

To further verify the predicted mechanism of network pharmacology, transcriptome analysis was performed and revealed 387 differentially expressed genes (DEGs) between the control and SQWCF groups, as shown in Fig. 5A–B, including 133 up-regulated and 254 down-regulated genes. Subsequently, KEGG analysis on the DEGs indicated significant enrichment in pathways in cancer, oxidative phosphorylation, PI3K-AKT signaling pathway, MAPK signaling pathway, and transcriptional misregulation in cancer (Fig. 5C). GO enrichment mainly included oxidoreductase activity, kinase binding, response to hormone, monoatomic cation transport, mitochondrial membrane, and centrosome (Fig. 5D). Furthermore, GSEA on the DEGs observed the activation or inactivation of typical signaling pathways in cancer cells following SQWCF interference. Specifically, SQWCF effectively inhibited the mRNA expressions of genes involved in various pathways, including cell cycle, central carbon metabolism, ubiquitin mediated proteolysis, and MAPK signaling (Fig. 5E–F).

### Identification of proteins and pathways related to the effect of SQWCF against GC by proteomic analysis

**Fig. 6:**
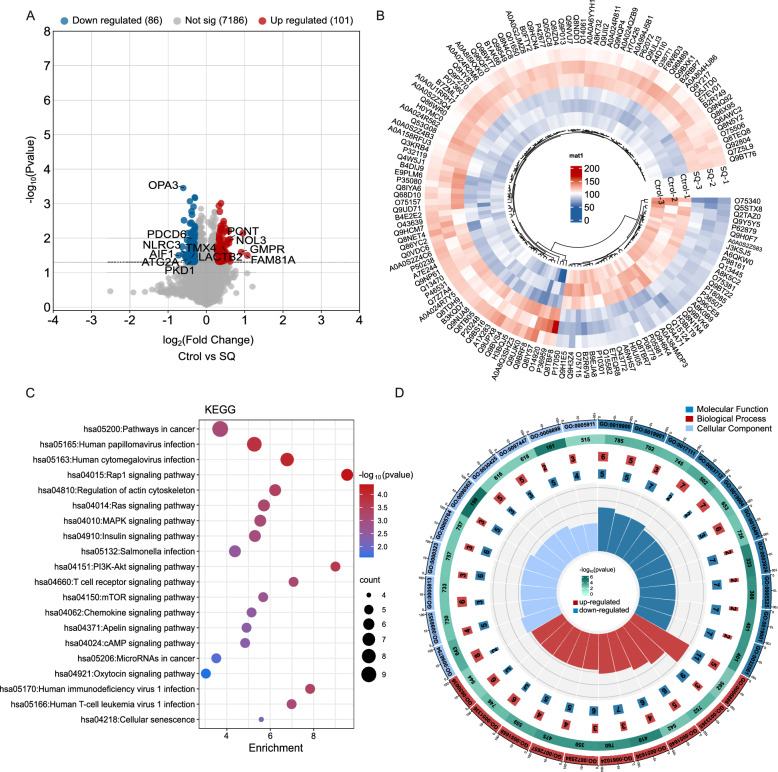
Proteomics analysis for the mechanisms of SQWCF against GC. **A** Volcano plot of DEPs between control and SQWCF group. **B** Heatmap of the different abundance proteins among control and SQWCF group. **C–D** KEGG and GO enrichment analysis of DEPs

To decipher the mechanism of SQWCF intervention in GC progression, proteomic analysis on tumor tissues treated with or without SQWCF were conducted and identified 187 differentially expressed proteins (DEPs) between the control and SQWCF groups, consisting of 101 up-regulated and 86 down-regulated proteins (Fig. 6A–B). KEGG enriched DEPs in pathways in cancer, Rap1 signaling pathway, regulation of actin cytoskeleton, Ras signaling pathway, and MAPK signaling pathway (Fig. 6C). GO analysis revealed that these DEPs were mainly enriched in kinase binding, protein kinase binding, postsynapse, side of membrane, intracellular protein transport, and protein localization to organelle (Fig. 6D).

### Inhibitory role of SQWCF in FAM81A expression at mRNA and protein levels

**Fig. 7:**
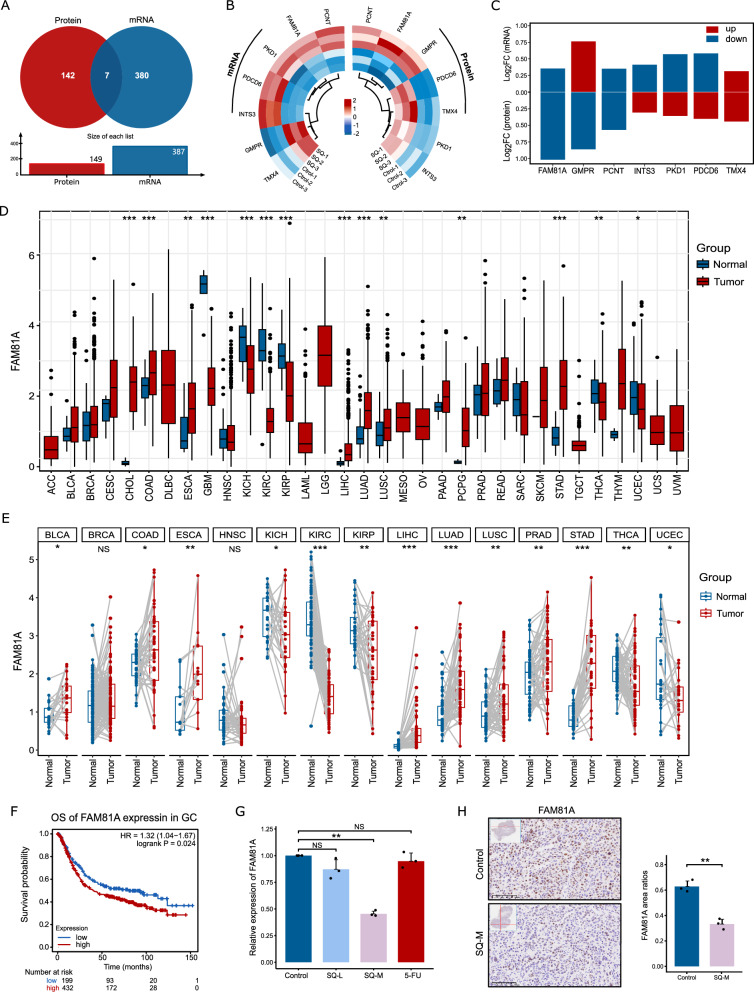
Analysis of transcriptomics combined with proteomics. **A** The common targets of DEGs and DEPs. **B** Heatmap visualizing targets that are differentially expressed in transcriptomics and proteomics analysis. **C** The fold change of the common differentially expressed targets in transcriptomics and proteomics analysis. **D** FAM81A expression in TCGA tumors and normal tissues with the data of the GTEx database as controls. **E** FAM81A expression in TCGA tumors and adjacent normal tissues. **F–I** The Kaplan–Meier survival curves of high and low FAM81A expression in GC through the Kaplan–Meier plotter database. **(J)** The mRNA expression of FAM81A in tumor tissues. **K** IHC staining of FAM81A in tumor tissues and statistical analysis of staining intensity. Data are presented as mean ± SD. *p < 0.05, **p < 0.01, ***p < 0.001, NS, no significant

Next, an intersection-based analysis of the DEGs and DEPs were employed to uncover the effect targets of SQWCF in treating GC. A total of 7 common targets of DEGs and DEPs were identified and shown in Fig. 7A. Specifically, FAM81A, PCNT, and TMX4 showed consistent changes at both mRNA and protein levels, with TMX4 being up-regulated, while FAM81A and PCNT being down-regulated (Fig. 7B–C). Subsequently, gene expression differential analysis and survival analysis were used to evaluate the value of these three genes in the diagnosis of cancer. There were significant differences in FAM81A, PCNT, and TMX4 expressions between tumor tissues and normal tissues in multiple types of cancer based on TCGA and GTEx. Notably, all three genes were aberrantly high expressed in GC tissues (Fig. 7D, Additional file 3: Fig. S3A, D). The paired sample analysis using TCGA database-sourced data showed the same noteworthy increase in the expressions of FAM81A and PCNT in cancers compared to paracancerous tissues (Fig. 7E, Additional file 3: Fig. S3B, E). Our subsequent experiment evaluated the prognostic value of FAM81A, PCNT, and TMX4 in overall survival (OS) in GC. High expression of FAM81A was significantly associated with poor prognosis for GC patients (Fig. 7F–I). However, the expression of PCNT and TMX4 showed no significant association with the prognosis of GC patients (Additional file 2: Fig. S2C-F, I-L).

To further verify the effect of SQWCF on FAM81A, the expression level of it was detected in the tumor issues via Q-PCR and IHC experiments. SQWCF suppressed the mRNA and protein levels of FAM81A in tumor tissues, consistent with omics data (Fig. 4 J–K). Moreover, the target FAM81A was stably docked with four compounds, of Glycitein, Liquiritigenin, Hesperetin, and Eriodictyol, supporting some potential interactions between FAM81A and these compounds (Additional file 3: Fig. S3C). With respect to the above, bioinformatics analyses supported FAM81A, a novel member of the pan-oncogene, was abnormally highly expressed and associated with poor prognosis in GC. FAM81A might exert a potential effect on the pathogenesis and progression of GC. In addition, SQWCF could uniformly inhibit FAM81A expression at both mRNA and protein levels, indicating that FAM81A could be recognized as a critical effector target of SQWCF to exert anti-GC activity of SQWCF.

### Effect of SQWCF on inhibiting the PI3K-AKT and MAPK signaling pathways and inducing downstream apoptosis pathway

**Fig. 8:**
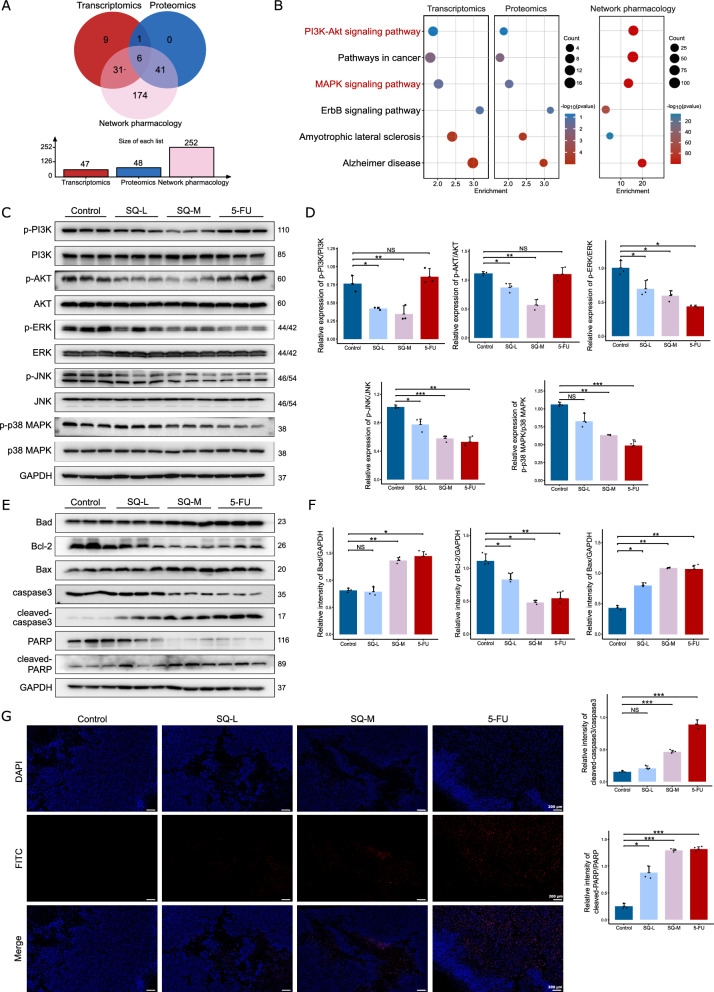
SQWCF inhibited the PI3K-AKT and MAPK signaling pathways and induced downstream apoptosis pathway. **A–B** The common enrichment pathways between network pharmacology, transcriptomics, and proteomics. **C–D** Western blot analysis of the protein expression levels of p-PI3K/PI3K, p-AKT/AKT, p-ERK/ERK, p-JNK/JNK, and p-p38 MAPK/p38 MAPK. **E–F** Western blot analysis of the protein expression levels of Bad, Bcl-2, Bax, caspase3, cleaved-caspase3, PARP, and cleaved-PARP. **G** TUNEL staining of the tumor tissues. Data are presented as mean ± SD. *p < 0.05, **p < 0.01, ***p < 0.001, NS, no significant

In order to comprehensively explore the underlying critical mechanism of SQWCF against GC, the enriched pathways of network pharmacology were integrated with transcriptomics and proteomics (Fig. 8A–B). Six pathways showed significant enrichment in both network pharmacology and omics analysis, among which PI3K-AKT and MAPK pathways were closely related to the development of tumors [16, 22]. Therefore, we hypothesized that these pathways might play key roles in the anti-GC effect of SQWCF.

**Fig. 9:**
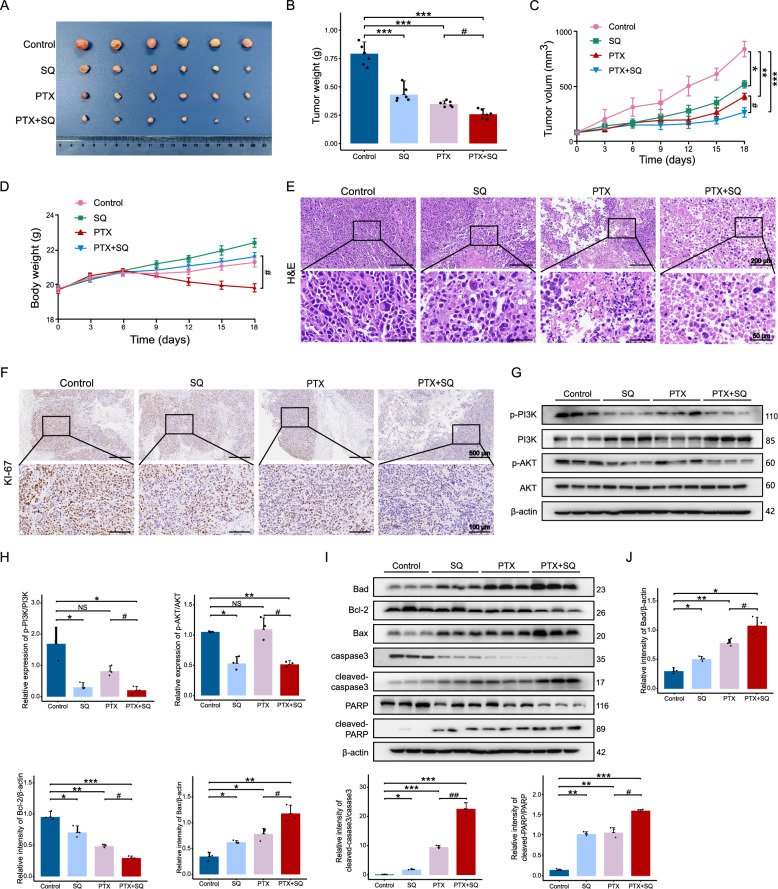
SQWCF combined with paclitaxel inhibited the growth of GC in vivo. **A** The physical appearance and size of the tumor tissues. **B** The tumor weight statistics. **C** The tumor volume statistics. **D** The body weight statistics. **E** H&E staining of the tumor tissues. **F** IHC staining of Ki-67 in tumor tissues. **G–H** Western blot analysis of the protein expression levels of p-PI3K/PI3K and p-AKT/AKT. **I–J** Western blot analysis of the protein expression levels of Bad, Bcl-2, Bax, caspase3, cleaved-caspase3, PARP, and cleaved-PARP. Data were presented as mean ± SD. Compared to control: *p < 0.05, **p < 0.01, ***p < 0.001, NS, no significant. Compared to PTX: ^#^*p* < 0.05, NS, no significant

To test our hypothesis, we assessed the expression of relative proteins in tumor tissues, as well as their phosphorylated proteins. In Fig. 8C–D, SQWCF significantly suppressed the expressions of p-PI3K/PI3K, p-AKT/AKT, p-ERK/ERK, p-JNK/JNK, and p-p38 MAPK/p38 MAPK, suggesting that SQWCF effectively suppressed PI3K-AKT and MAPK pathways. Furthermore, apoptosis is a kind of programmed cell death targeted by most anticancer drugs [32, 33], which can be mediated by PI3K-AKT and MAPK pathways [34]. Therefore, markers of apoptosis (Bcl-2, Bax, Bad, caspase3, cleaved-caspase3, PARP, and cleaved-PARP) were detected to investigate the activation of apoptosis pathway. In Fig. 9E–F, SQWCF significantly increased Bad, Bax, cleaved-caspase3, and cleaved-PRAP expressions, and decreased Bcl-2, PARP, and caspase3. indicating the initiation of apoptosis pathway. Meanwhile, there was a significant decrease in TUNEL-positive cells following SQWCF treatment (Fig. 9G). As a positive control, 5-FU also significantly promoted apoptosis (Fig. 9C–G). Taken together, SQWCF effectively inhibited the activation of PI3K-AKT and MAPK pathways, subsequently promoting downstream apoptotic signal transduction.

### Synergistic effect of SQWCF combined with PTX on inhibiting GC in vivo

PTX is a widely used chemotherapeutic agent that is known to induce apoptosis in various cells [35, 36]. Abnormal activation of PI3K-AKT signaling pathway is one of the main reasons for PTX resistance [37, 38], adversely affecting its therapeutic efficacy. Considering that SQWCF could effectively inhibit the activity of PI3K-AKT signaling pathway. we employed a subcutaneous transplantation tumor model to dissect whether SQWCF combined with PTX had a synergistic effect. As shown in Fig. 9A–C, significant reductions in both tumor volume and weight were observed in treatment groups compared to the control group. Notably, the combined therapy showed an excellent anticancer effect, which was superior to SQWCF or PTX alone. Consistent results were also obtained in H&E staining (Fig. 9E). Moreover, in the combined treatment group, SQWCF alleviated PTX-induced weight loss (Fig. 9D). In addition, SQWCF markedly inhibited the phosphorylation of both PI3K and AKT (Fig. 9F–G), consistent with aforementioned findings. There was no significant change in the activity of PI3K-AKT signaling pathway following paclitaxel treatment. However, this pathway was markedly inhibited after adding SQWCF. Additionally, expression levels of apoptosis-related proteins Bax and Bad were increased, while caspase-3 and Bcl-2 were decreased in treatment groups compared to the control group (Fig. 9H–I). As expected, combined treatment resulted in stronger apoptotic signals than SQWCF or PTX alone. These findings supported a synergistic anticancer effect of SQWCF and PTX, which may be related to the suppression of PI3K-AKT pathway.

## Discussion

As a representative digestive tract malignancy, GC is featured by high heterogeneity and poor prognosis due to lack of early diagnosis [39]. Chemotherapy, molecularly targeted therapy, and immunotherapy represented by ICIs can prolong the OS of patients with GC, yet with limited efficacy due to drug resistance, toxicity, and side-effect [4, 5]. So far, there is an urgent need to develop a novel anticarcinogen that can provide long-term benefits for all patients with GC with low toxicity. TCM has been widely used in treating cancer patients with promising prospect due to its high-efficiency anticancer activity, low drug resistance, and little toxic and side effects [8, 40]. SQWCF is a patented traditional Chinese herbal formula for GC, while it is still unclear with regard to the efficacy and underlying mechanism. In this study, we confirmed that SQWCF effectively inhibited the growth of GC in vivo without obvious side effects and comprehensively revealed the synergistic regulatory mechanism of the multi-component, multi-target, and multi-pathway of SQWCF in treating GC.

Initially, we constructed a biomolecular network of SQWCF against GC through network pharmacology based on mass spectrometry to analyze the underlying complex workings. Network topology analysis identified 4 key gene targets (AKT1, BCL2, TP53, and CASP3) along with 5 critical active compounds (Calycosin, Glycitein, Liquiritigenin, Hesperetin, and Eriodictyol) of SQWCF relevant to GC treatment. The stable target-compound binding affinity was further validated using molecular docking. Notably, AKT1 emerged as the gene target exhibiting the strongest affinity. Furthermore, KEGG and GO analysis demonstrated the common targets shared between SQWCF and GC were significantly enriched in multiple tumor-associated biological pathways, including PI3K-AKT and MAPK signaling pathways, and involved in the biological processes of “phosphorylation” and “protein phosphorylation”. These 5 core active compounds have been proven to have powerful anticancer effects by inhibiting PI3K-AKT or MAPK signaling pathway. Specifically, Calycosin and Hesperetin could retard the activation of PI3K-AKT pathway, thereby inhibiting triple-negative breast cancer development and metastasis [41, 42]. Glycitein was reported as a potential targeted therapeutic agent for human GC by inducing apoptosis via MAPK-STAT3-NF-kB signaling pathway [43]. Similarly, Liquiritigenin could suppress oral cancer progression through inducing autophagy-associated apoptosis via PI3K-AKT-mTOR pathway [44]. Research by He et al. showed that Eriodictyol also exerted an inhibitory effect on breast carcinogenesis by repressing PI3K-AKT pathway [45]. All these studies strongly support our results of analysis. Therefore, it is possible that Calycosin, Glycitein, Liquiritigenin, Hesperetin, and Eriodictyol could be accepted as effective therapeutic components of SQWCF for preventing GC by repressing PI3K-AKT and MAPK pathways. However, the core active compounds of SQWCF are mainly based on network topology analysis and lack of experimental research in vivo. Thus, pharmacological research of SQWCF should be investigated and how these compounds coordinated with each other should be revealed in the future study.

In further exploration, transcriptomic and proteomic profiles were integrated to elucidate the DEGs and DEPs regulated by SQWCF. Enrichment analysis of DEGs or DEPs supported the potential anticancer effects of SQWCF considering the enrichment of multiple biological processes and pathways in cancer. Importantly, PI3K-AKT and MAPK pathways were significantly enriched in this analysis, aligning with the network pharmacological predictions. Therefore, the modulation of PI3K-AKT and MAPK pathways may be the key mechanism for elucidating the anti-GC role of SQWCF. Moreover, PI3K-AKT and MAPK pathways have been reported widely for their roles of promoting cancer formation and promotion [17, 20, 23], highlighting their potential as targets for novel anti-tumor strategies. In our study, SQWCF was confirmed to hinder both PI3K-AKT and MAPK signaling pathways and boost downstream apoptotic signaling, suggesting that SQWCF may play an anti-GC role as an inhibitor of PI3K-AKT and MAPK signaling pathways.

In addition, intersection-based analysis on the screened DEGs and DEPs identified that FAM81A was significantly down-regulated at both mRNA and protein levels after SQWCF treatment. Q-PCR, immunohistochemistry, along with molecular docking studies further corroborated the regulatory effect of SQWCF on FAM81A, suggesting that FAM81A could serve as a potential effector target for SQWCF. FAM81A belongs to the family with sequence similarity 81, with the highest protein expression level in the adult brain, and its specific function remains unknown [46]. FAM81A is recently reported to have a potential role in the occurrence and development of tumor. Guo et al. discovered that FAM81A could regulate the tumor microenvironment in pancreatic cancer, as a protein-coding gene that with close relationship to cellular processes and pathways such as cell cycle, differentiation, and DNA repair [47]. Yamamoto et al. showed that CNVs in FAM81A and PCSK6 were significantly associated with the recurrence and progression of noninvasive bladder cancer [48]. To date, the role of FAM81A in GC remains unexplored. Our research for the first time indicated that FAM81A was abnormally overexpressed and associated with poor prognosis in GC, showing its potential utility as a biomarker to guide the treatment of GC, providing new insights into the development of therapeutic approaches for GC. However, our clarification of FAM81A was mainly based on data harvested public database mining, warranting further validation in a clinically independent cohort. Additionally, further study is required to validate the pharmaceutical active components targeting FAM81A of SQWCF and the mechanism of FAM81A involved in the anti-GC effects of SQWCF.

Another significant finding of this study was that SQWCF exhibited a synergistic effect with PTX in treating GC in vivo. PTX is a widely used natural anticancer drug [49], and is already approved for the treatment of advanced GC [50]. However, resistance to PTX poses a great challenge for its expanded application [51]. Increasing evidence suggests that cancer progression and chemoresistance are coupled with the abnormal activation of PI3K-AKT signaling pathway [37]. Thus, the combination of drugs targeting PI3K-AKT signaling pathway represents an effective strategy to overcome the resistance to PTX [38, 52]. In this study, SQWCF and PTX could exert a synergistic anti-GC effect by promoting apoptosis. As expected, the application of SQWCF could downregulate the PI3K-AKT pathway, indicating that the proposed synergistic effect might be associated with the regulation of the PI3K-AKT pathway. Anyway, further exploration is required to confirm whether PI3K-AKT pathway may be involved in the synergistic inhibitory effect of SQWCF and PTX. SQWCF exhibited a potential inhibitory role in PI3K-AKT pathway activation. which may be an effective drug to treat GC in combination with PTX or other drugs that cause resistance due to abnormal activation of PI3K-AKT pathway. TCM-based adjuvant chemotherapy for anticancer therapies has been highly concerned in current research of cancers [8]. Therefore, our discovery will shed light on drug development based on the inhibition of PI3K-AKT pathway and provide a clue for developing adjunctive drugs to overcome chemoresistance in GC therapy.

## Conclusion

In this study, we reported that SQWCF, which is a patented traditional herbal prescription for clinical GC treatment, could suppress GC progression with minimal toxicity and enhance therapeutic efficacy when combined with PTX. Mechanically, SQWCF inhibited the activation of both PI3K-AKT and MAPK signaling pathways to induce apoptotic signal transduction. In addition, our study for the first time discovered that FAM81A is an effector target for SQWCF to treat GC. In summary, SQWCF is a potentially effective complementary and alternative therapy in the treatment of GC.

## Supplementary Information

Additional file 1 (PDF 638 kb)

Additional file 2 (PDF 9561 kb)

Additional file 3 (PDF 9560 kb)

Additional file 4 (DOCX 70 kb)

## Data Availability

The data associated with this study can be obtained from the corresponding author upon reasonable request.

## Abbreviations

AKT: Protein kinase B
AKT1: AKT serine/threonine kinase 1
ALT: Alanine aminotransferase
AST: Aspartate aminotransferase
Bad: Bcl-2 associated agonist of cell death
Bax: Bcl-2-associated X
Bcl-2: B-cell lymphoma 2
CREA: Creatinine
ERK: Extracellular-signal-regulated kinase
FAM81A: Family with sequence similarity 81 member A
GC: Gastric cancer
GO: Gene ontology
GSEA: Gene set enrichment analysis
H&E: Hematoxylin and eosin
IHC: Immunohistochemical
JNK: C-Jun N-terminal kinase
KEGG: Kyoto encyclopedia of genes and genomes
Ki-67: Kiel 67
MAPK: Mitogen-activated protein kinase
PARP: Nuclear poly (ADP-ribose) polymerase
PIP3: Phospha-tidylinositol (3,4,5) trisphosphates
PI3K: Phosphoinositide 3-kinase
PPI: Protein and protein interaction
PTX: Paclitaxel
Q-PCR: Quantitative real-time PCR
SQWCF: Shen Qing Weichang formula
TCM: Traditional chinese medicine
TUNEL: TdT-mediated dUTP nick-end labeling
UREA: Carbonyl diamide
5-FU: 5-Fluorouracil
