# Supplementary figures and images for: Integrative network pharmacology, transcriptomics, and proteomics reveal the material basis and mechanism of the Shen Qing Weichang Formula against gastric cancer
Source: Chin Med. 2025 Mar 29;20:42. doi: 10.1186/s13020-025-01091-4 (PMC11954191; doi:10.1186/s13020-025-01091-4)

A

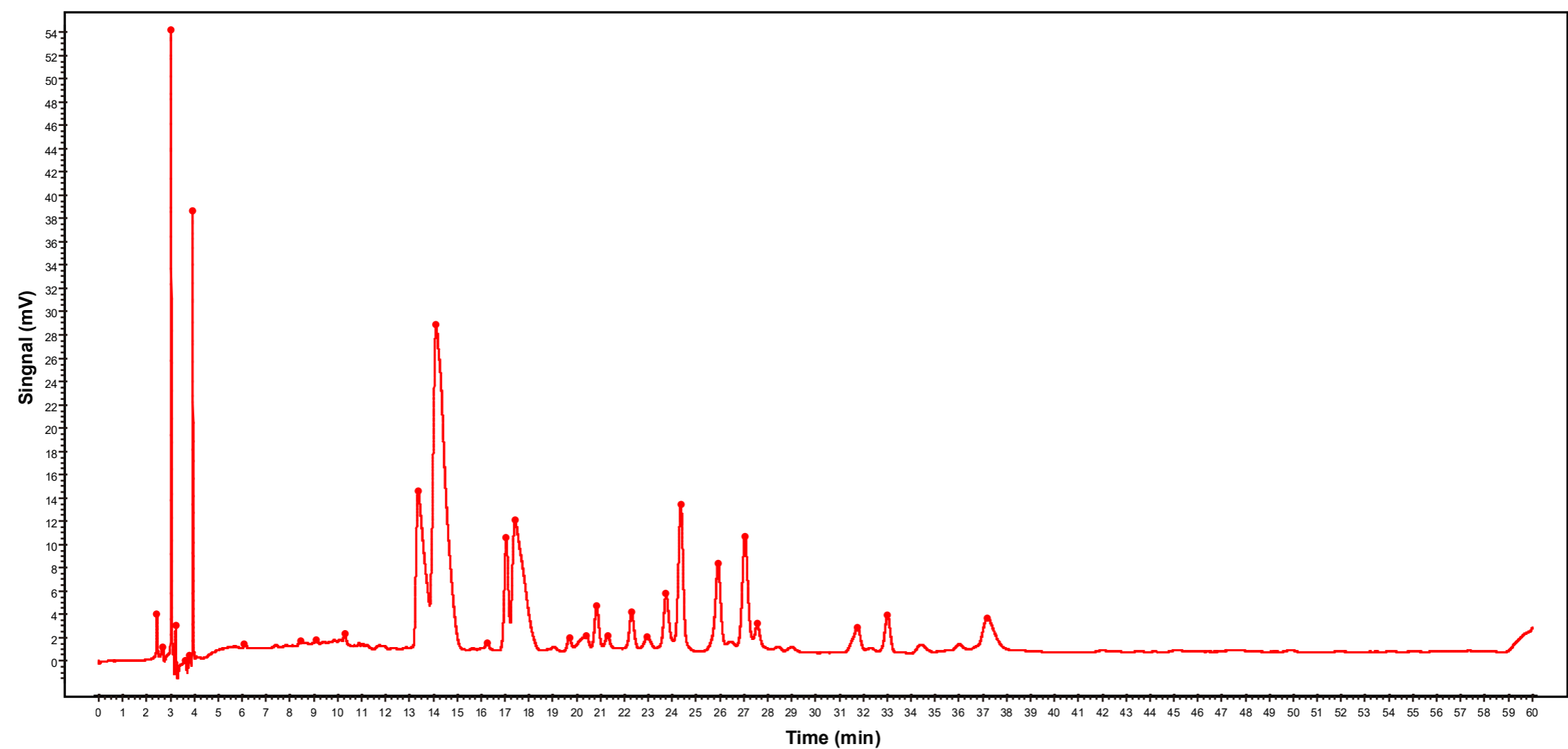

B

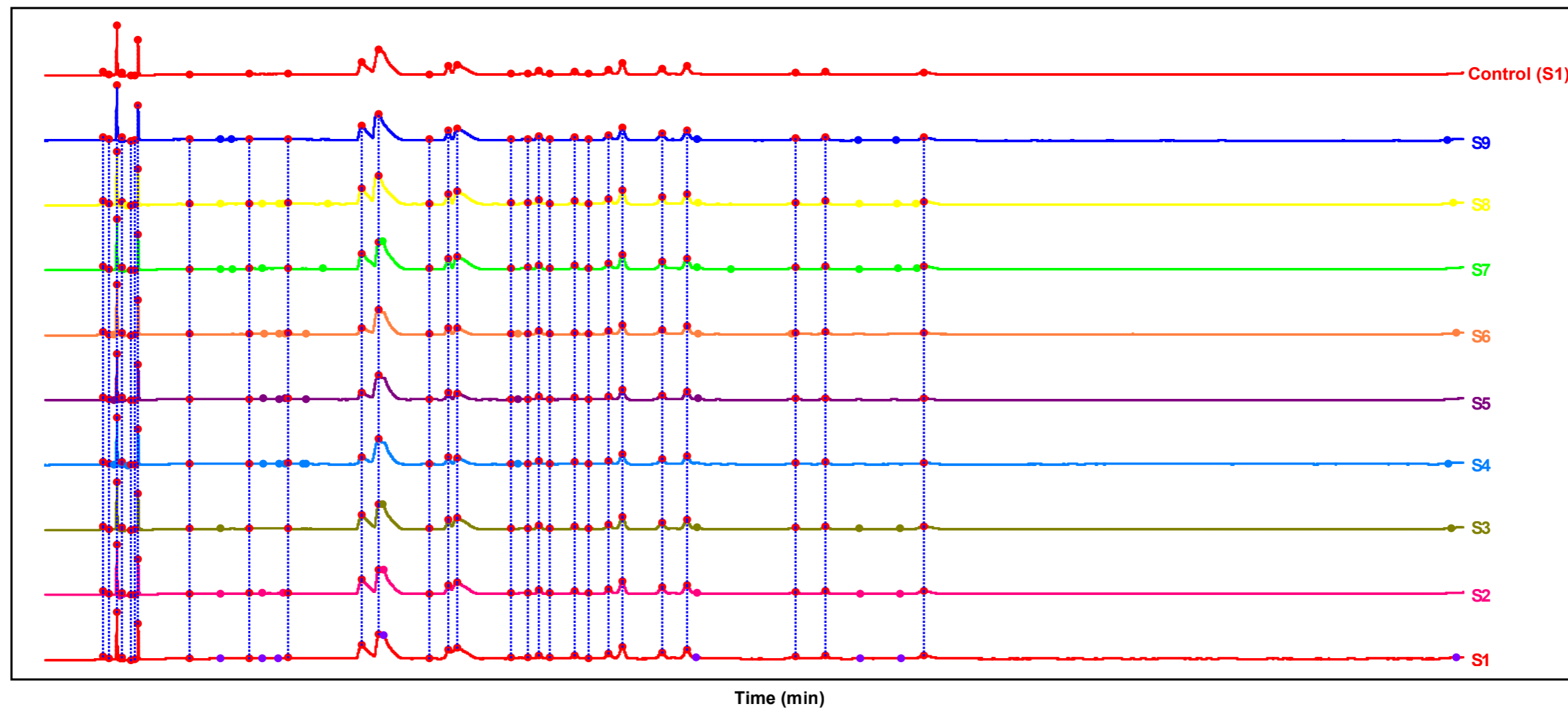

Supplement: Supplementary file 1 — Additional file 1 (PDF 638 kb) [file 13020_2025_1091_MOESM1_ESM.pdf]
