# Supplementary material for: Integrative network pharmacology, transcriptomics, and proteomics reveal the material basis and mechanism of the Shen Qing Weichang Formula against gastric cancer
Source: Chin Med. 2025 Mar 29;20:42. doi: 10.1186/s13020-025-01091-4 (PMC11954191; doi:10.1186/s13020-025-01091-4)
Supplement: Supplementary file 2 — Additional file 2 (PDF 9561 kb) [file 13020_2025_1091_MOESM2_ESM.pdf]

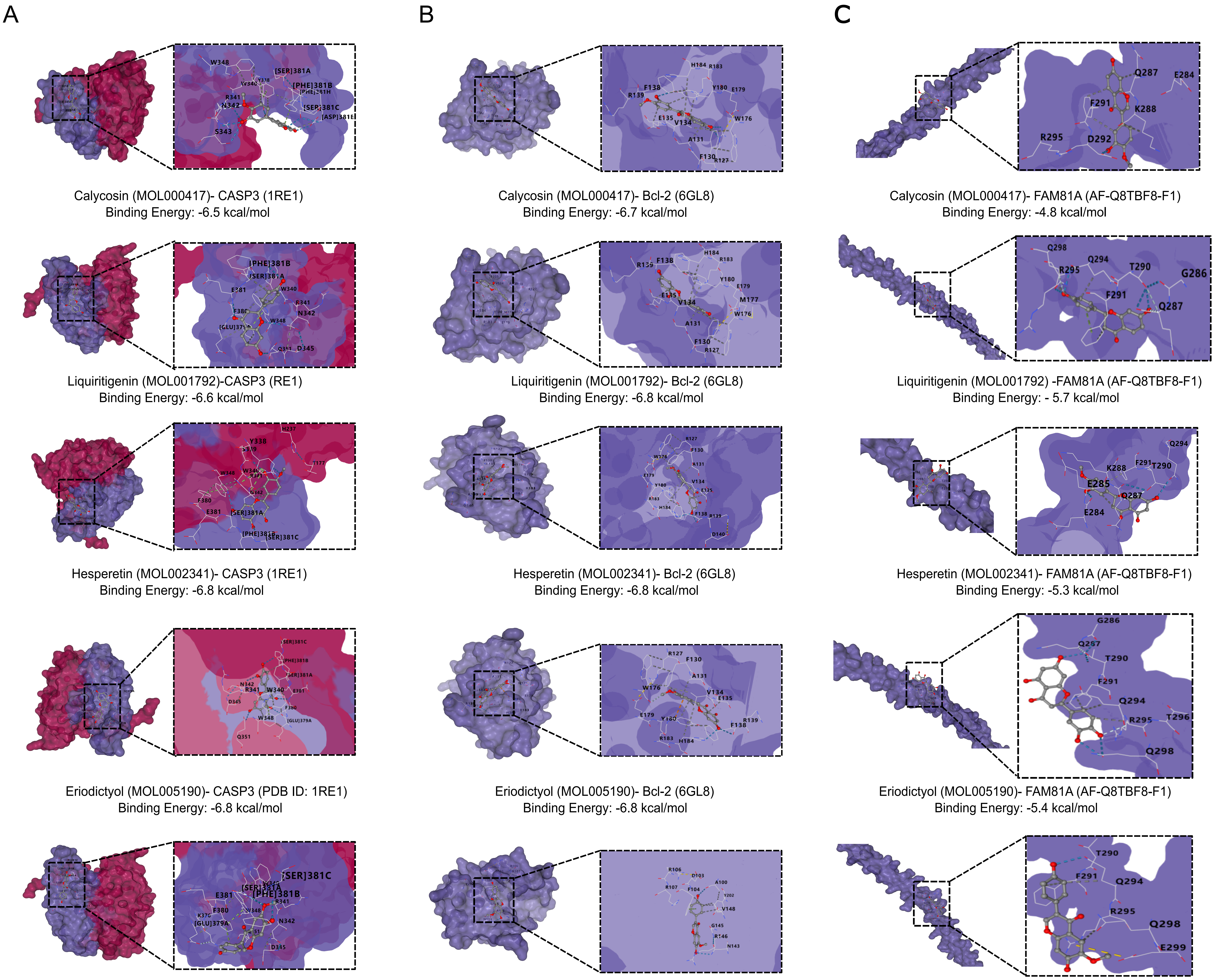

Calycosin (MOL000417)- CASP3 (1RE1)
Binding Energy: -6.5 kcal/mol

Calycosin (MOL000417)- Bcl-2 (6GL8)
Binding Energy: -6.7 kcal/mol

Calycosin (MOL000417)- FAM81A (AF-Q8TBF8-F1)
Binding Energy: -4.8 kcal/mol

Liquiritigenin (MOL001792)-CASP3 (RE1)
Binding Energy: -6.6 kcal/mol

Liquiritigenin (MOL001792)- Bcl-2 (6GL8)
Binding Energy: -6.8 kcal/mol

Liquiritigenin (MOL001792) -FAM81A (AF-Q8TBF8-F1)
Binding Energy: - 5.7 kcal/mol

Hesperetin (MOL002341)- CASP3 (1RE1)
Binding Energy: -6.8 kcal/mol

Hesperetin (MOL002341)- Bcl-2 (6GL8)
Binding Energy: -6.8 kcal/mol

Hesperetin (MOL002341)- FAM81A (AF-Q8TBF8-F1)
Binding Energy: -5.3 kcal/mol

Eriodictyol (MOL005190)- CASP3 (PDB ID: 1RE1)
Binding Energy: -6.8 kcal/mol

Eriodictyol (MOL005190)- Bcl-2 (6GL8)
Binding Energy: -6.8 kcal/mol

Eriodictyol (MOL005190)- FAM81A (AF-Q8TBF8-F1)
Binding Energy: -5.4 kcal/mol

Glycitein (MOL008400)- CASP3 (PDB ID: 1RE1)
Binding Energy: -6.2 kcal/mol

Glycitein (MOL008400)- Bcl-2 (PDB ID: 6GL8)
Binding Energy: -6.3 kcal/mol

Glycitein (MOL008400)- FAM81A (AF-Q8TBF8-F1)
Binding Energy: -5.2 kcal/mol
