# Supplementary material for: Integrative network pharmacology, transcriptomics, and proteomics reveal the material basis and mechanism of the Shen Qing Weichang Formula against gastric cancer
Source: Chin Med. 2025 Mar 29;20:42. doi: 10.1186/s13020-025-01091-4 (PMC11954191; doi:10.1186/s13020-025-01091-4)
Supplement: Supplementary file 3 — Additional file 3 (PDF 9560 kb) [file 13020_2025_1091_MOESM3_ESM.pdf]

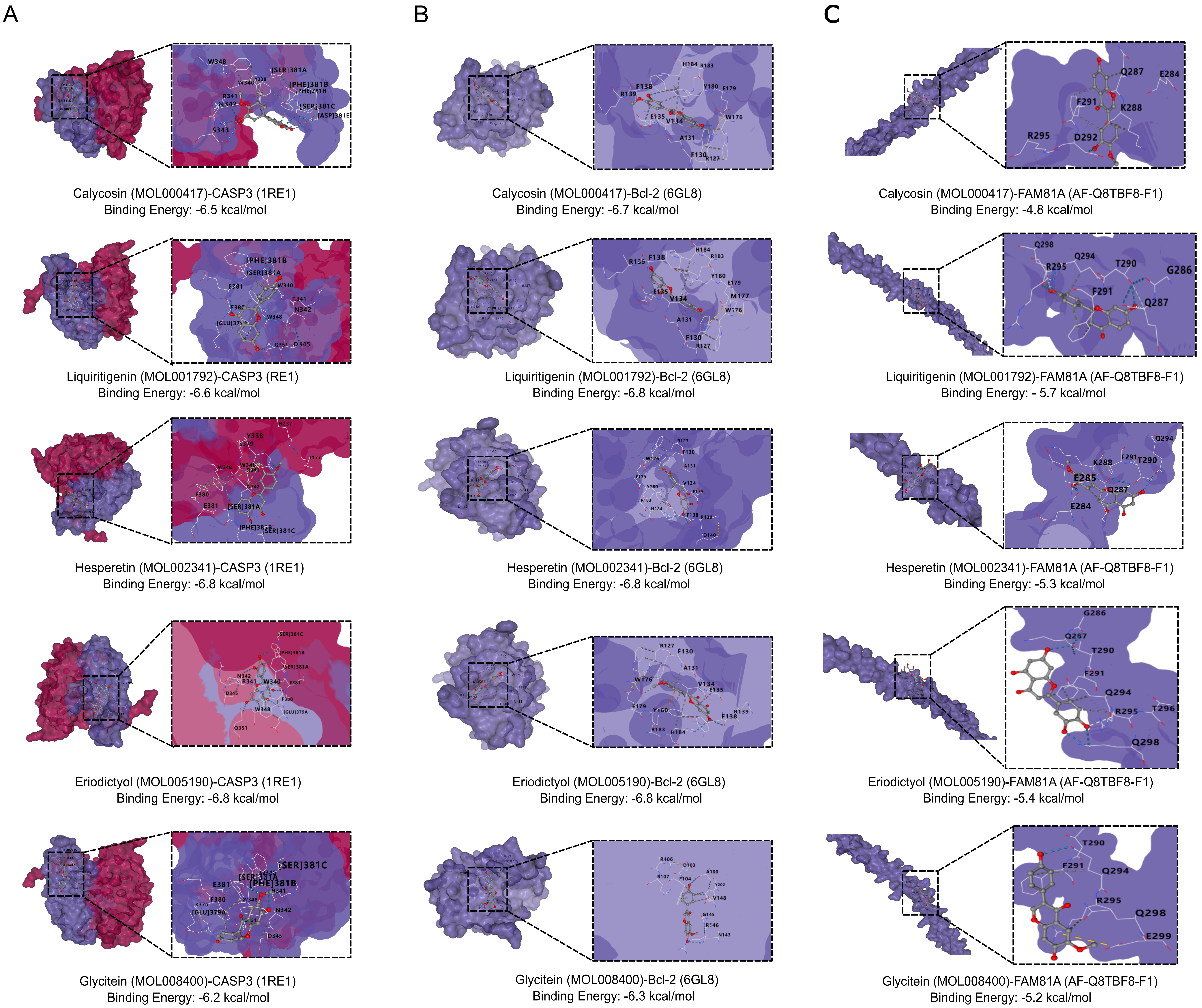

A
B
C
Calycosin (MOL000417)-CASP3 (1RE1)
Binding Energy: -6.5 kcal/mol
Calycosin (MOL000417)-Bcl-2 (6GL8)
Binding Energy: -6.7 kcal/mol
Calycosin (MOL000417)-FAM81A (AF-Q8TBF8-F1)
Binding Energy: -4.8 kcal/mol
Liquiritigenin (MOL001792)-CASP3 (RE1)
Binding Energy: -6.6 kcal/mol
Liquiritigenin (MOL001792)-Bcl-2 (6GL8)
Binding Energy: -6.8 kcal/mol
Liquiritigenin (MOL001792)-FAM81A (AF-Q8TBF8-F1)
Binding Energy: - 5.7 kcal/mol
Hesperetin (MOL002341)-CASP3 (1RE1)
Binding Energy: -6.8 kcal/mol
Hesperetin (MOL002341)-Bcl-2 (6GL8)
Binding Energy: -6.8 kcal/mol
Hesperetin (MOL002341)-FAM81A (AF-Q8TBF8-F1)
Binding Energy: -5.3 kcal/mol
Eriodictyol (MOL005190)-CASP3 (1RE1)
Binding Energy: -6.8 kcal/mol
Eriodictyol (MOL005190)-Bcl-2 (6GL8)
Binding Energy: -6.8 kcal/mol
Eriodictyol (MOL005190)-FAM81A (AF-Q8TBF8-F1)
Binding Energy: -5.4 kcal/mol
Glycitein (MOL008400)-CASP3 (1RE1)
Binding Energy: -6.2 kcal/mol
Glycitein (MOL008400)-Bcl-2 (6GL8)
Binding Energy: -6.3 kcal/mol
Glycitein (MOL008400)-FAM81A (AF-Q8TBF8-F1)
Binding Energy: -5.2 kcal/mol
