# Supplementary material for: Integrative network pharmacology, transcriptomics, and proteomics reveal the material basis and mechanism of the Shen Qing Weichang Formula against gastric cancer
Source: Chin Med. 2025 Mar 29;20:42. doi: 10.1186/s13020-025-01091-4 (PMC11954191; doi:10.1186/s13020-025-01091-4)
Supplement: Supplementary file 4 — Additional file 4 (DOCX 70 kb) [file 13020_2025_1091_MOESM4_ESM.docx]

**Supplementary Materials**

**Supplementary materials and methods**

**Quality control research of SQWCF**

**Preparation of SQWCF** **working solution**

1 g of sample were dissolved in 3 ml methanol, treated with ultrasound (180 W，45 kHz，20 ℃ ) for 10 min, centrifuged for 10 min at 14 000 r/min and then filtrated by a microporous filter membrane (0.45 μm) to obtain the working solution. Following the same procedure as above, working solution from different batches of SQWCF was transferred into a sampling bottle for fingerprint analysis by HPLC.

**Conditions of chromatogram**

We used a ACQUITY UPLC^®^ HSS T3 chromatographic column (1.7 µm, 2.1 mm X 100 mm, Waters, USA). A mobile phase contains water and 0.1% formic acid; B is acetonitrile and 0.1% formic acid, with an elution gradient of 0-2 min, 2-5% B; 2-10 min, 5-2% B; 10-15 min, 15-25% B; 15-18 min, 25-50% B; 18-23 min, 50-100% B; 23-30 min, 100-2% B; Column temperature 40 ℃, flow rate 0.3 ml/min, sample chamber temperature 4 ℃.

**Establishment of fingerprint**

After testing for repeatability, accuracy, and stability, SQWCF granule decoction S1-S6 was used to prepare working solution and its fingerprint was determined and recorded according to the described chromatographic conditions. The data were imported into the similarity evaluation system for chromatographic fingerprint of TCM (v 2012), and S1 was set as the reference spectrum and the time window width was set to 0.1 min. After multi-point correction, the mark peak matching was performed to obtain the HPLC superposed fingerprint and corresponding control fingerprint, as shown in Fig S1.

**Evaluation of similarity**

According to the systematic evaluate of the similarity evaluation system, the results showed that the similarity of 6 batches was greater than 0.900, indicating that most of the samples had small differences and good quality stability, as shown in Table S1.

**Transcriptomics study**

**RNA sample quantification**

Nanodrop^TM^ 2000 spectrophotometer (Thermo Scientific, USA) was used to determine the concentration and evaluate the purity of RNA samples. Agilent 2100 Bioanalyzer and 2100 RNA nano 6000 assay kit (Agilent, USA) were used to evaluate the integrity of RNA samples.

**Transcriptome sequencing library preparation**

After the QC procedure, the RNA with poly-A in eukaryotic total RNA was enriched by TIANSeq mRNA Capture Kit (TIANGEN, Beijing). Then, using the captured RNA as the starting sample, TIANSeq Fast RNA Library Kit (Illumina, USA) was used to construct the transcriptome sequencing libraries. Briefly, the transcriptome sequencing library was constructed through RNA randomly fragmentation, cDNA strand 1/2 strand synthesis, end repair, A-tailing, ligation of sequencing adapters, size selection and library PCR enrichment.

**Library quantification**

Library concentration was first quantified using Qubit 2.0 fluorometer (Life Technologies, USA), and then diluted to 1 ng/µL before checking insert size on an Agilent 2100 Bioanalyzer (Agilent, USA) and quantifying to greater accuracy by quantitative PCR (Q-PCR) (library activity >2 nM).

**Clustering and sequencing**

The clustering of the index-coded samples was performed on a cBot Cluster Generation System using TruSeq PE Cluster Kit v3-cBot-HS (Illumina, USA) according to the manufacturer’s instructions. After cluster generation, the library preparations were sequenced on an Illumina sequencing platform and 150 bp paired-end reads were generated.

**Proteomics study**

**Total protein extraction**

Take out the samples in the frozen state and put it on ice. Add an appropriate amount of protein lysate (8 M urea, 1% SDS), which contains protease inhibitor to inhibit protease activity. The mixture was treated by ultrasound for 2 min at a low temperature, following splitting for 30 min. After centrifugation at 12, 000 g at 4°C for 30 min, the concentration of protein supernatant was determined by Bicinchoninic acid (BCA) method by BCA Protein Assay Kit（(Thermo Scientific, USA). Protein quantification was performed according to the kit protocol.

**Protein reductive alkylation and digestion**

Take protein samples 100 μg and add triethylammonium bicarbonate buffer (TEAB), which the final concentration of TEAB is 100 mM. Then add tris (2-carboxyethyl) phosphine (TCEP) to the final concentration of 10 mM and react for 60 min at 37 °C. Following add Iodoacetamide (IAM) to the final concentration of 40 mM and react for 40 min at room temperature under dark conditions. Add a certain percentage (acetone: sample v/v = 6:1) of pre-cooled acetone to each sample and to settle for 4 h at -20 °C. After centrifugal for 20 min at 10,000 g, the sediment was collected and add 100 µL 100 mM TEAB solution to dissolve. Finally, the mixture was digested with Trypsin overnight at 37 °C added at 1:50 trypsin-to-protein mass ratio.

**Peptide desalination**

The peptides were vacuum dried, then resuspended with 0.1% Triethylammonium bicarbonate buffer (TFA). Samples were desalted with hydrophile-lipophile balance (HLB), and vacuum dried. Peptide concentrations were determined by Pierce^TM^ Quantitative Peptide Assays & Standards (Thermo Scientific, USA).

**TMT Labeling**

One unit of TMT reagent were thawed and reconstituted in acetonitrile. After tagging for 2 h at room temperature, hydroxylamine was added to react for 30 min at room temperature. In our study, the samples were labeled as corresponding identifier. Finally, all samples were pooled, desalted and vacuum-dried.

**High pH RPLC Separation**

The pooled samples were fractionated into fractions by Vanquish^TM^ Flex UHPLC Systems (Thermo Scientific, USA) with ACQUITY UPLC BEH C18 Column (1.7 µm, 2.1mm × 150 mm, Waters, USA) to increase proteomic depth. Briefly, peptides were first separated with a gradient of elution (Phase A: 2% acetonitrile, pH: 10; Phase B: 80% acetonitrile, pH: 10) over 48 min at a flowrate of 200 μL/min. The peptides were eluted using the following gradient: 0-1.9 min, 0-0% B; 1.9-2 min, 0-5% B; 2-17 min, 5-5% B; 17-18 min, 5-10% B; 18-35.5 min, 10-30% B; 35.5-38 min, 30-36% B; 38-39 min, 36-42% B; 39-40 min, 42-100% B; 40-44 min, 100-100% B; 44-45 min, 100-0% B; 45-48 min, 0-0% B. Twenty-eight fractions were collected from each sample, which was subsequently pooled, resulting in fourteen total fractions per sample.

**LC-MS/MS analysis**

Labeled peptides were analyzed by online nano flow liquid chromatography tandem mass spectrometry performed on an Evosep One system (Evosep, Denmark) connected to a Orbitrap Exploris 480 (Thermo Scientific, USA) through a nano-electrospray ion source. Briefly, the C18-reversed phase column (150 μm×15 cm, Evosep, Denmark) as equilibrated with solvent A (water with 0.1% formic acid) and solvent B (ACN with 0.1% formic acid).The peptides were eluted using the following gradient: 0-2 min, 5-5% B; 2-30 min, 5−38% B; 30-40 min, 38−90% B; 40−44 min, 90−90% B at a flow rate of 300 nL/min. The Orbitrap Exploris 480 was operated in the data-dependent acquisition mode (DDA) to automatically switch between full scan MS and MS/MS acquisition, meanwhile TurboTMT algorithm was applied. The survey of full scan MS spectra (m/z 350-1500) was acquired in the Orbitrap with resolution ratio of 60,000. Precursor ions were into collision cell for fragmentation by higher-energy collision dissociation (HCD). The MS/MS resolution was set at 15,000 resolution ratio (at fixed first mass m/z 110), minimum automatic gain control (AGC) target at 8e3, the maximum fill time at 22 ms, and dynamic exclusion was 30 seconds.

**Protein identification**

The raw data files were analyzed using ProteomeDiscoverer^TM^ (Thermo Scientific, v 3.0) against UniProt database. The MS/MS search criteria were as follows: precursor mass tolerance of 20 ppm for MS and fragment mass tolerance of 0.02 Da. False discovery rate (FDR) of peptide identification was set as FDR ≤ 0.01. A minimum of one unique peptide identification was used to support protein identification.

**Supplementary tables and legends**

**Table S1 Similarity evaluation of 6 branches of SQWCF**

| Sample number | Similarity score |
| --- | --- |
| Control (S1) | 1 |
| S2 | 0.931 |
| S3 | 0.921 |
| S4 | 0.913 |
| S5 | 0.915 |
| S6 | 0.932 |
| S7 | 0.934 |
| S8 | 0.993 |
| S9 | 0.998 |

**Table S2 Identification of the chemical constituents of SQWCF by UHPLC-MS/MS**

| No. | Compound | Molecular weight | Formula | T_R_ (min) |
| --- | --- | --- | --- | --- |
| 1 | Chlorogenic acid | 354.0952 | C_16_H_18_O_9_ | 5.499 |
| 2 | Choline | 103.09982 | C_5_H_13_NO | 0.806 |
| 3 | Berberine | 335.1158 | C_20_H_17_NO_4_ | 6.109 |
| 4 | Specnuezhenide | 686.24191 | C_31_H_42_O_17_ | 6.94 |
| 5 | Cryptochlorogenic acid | 354.09514 | C_16_H_18_O_9_ | 7.306 |
| 6 | Sucrose | 342.11621 | C_12_H_22_O_11_ | 0.843 |
| 7 | Mannitol | 182.07904 | C_6_H_14_O_6_ | 0.897 |
| 8 | (R)-Mandelic acid | 152.0473 | C_8_H_8_O_3_ | 5.345 |
| 9 | Adenosine | 267.09682 | C_10_H_13_N_5_O_4_ | 3.523 |
| 10 | Quinic acid | 192.06343 | C_7_H_12_O_6_ | 5.504 |
| 11 | Eleutheroside E | 742.26804 | C_34_H_46_O_18_ | 6.143 |
| 12 | 2-Pyrrolidinecarboxylic acid | 115.06352 | C_5_H_9_NO_2_ | 0.889 |
| 13 | Protocatechualdehyde | 138.03164 | C_7_H_6_O_3_ | 3.451 |
| 14 | Isoleucine | 131.0949 | C_6_H_13_NO_2_ | 1.364 |
| 15 | (+)-Catechin hydrate | 290.07914 | C_15_H_14_O_6_ | 3.542 |
| 16 | Erucamide | 337.33454 | C_22_H_43_NO | 15.41 |
| 17 | Adenine | 135.05475 | C_5_H_5_N_5_ | 1.47 |
| 18 | Rutin | 610.15336 | C_27_H_30_O_16_ | 6.397 |
| 19 | Isoquercitrin | 464.09578 | C_21_H_20_O_12_ | 6.595 |
| 20 | Heterophyllin B | 778.43688 | C_40_H_58_N_8_O_8_ | 9.349 |
| 21 | Pipecolic acid | 129.07925 | C_6_H_11_NO_2_ | 0.929 |
| 22 | Betaine | 117.07911 | C_5_H_11_NO_2_ | 0.963 |
| 23 | Morin | 302.04277 | C_15_H_10_O_7_ | 6.595 |
| 24 | L-Pyroglutamic acid | 129.04286 | C_5_H_7_NO_3_ | 2.969 |
| 25 | L-Phenylalanine | 165.07929 | C_9_H_11_NO_2_ | 2.552 |
| 26 | DL-Arginine | 174.11188 | C_6_H_14_N_4_O_2_ | 0.842 |
| 27 | α-Lactose | 342.11632 | C_12_H_22_O_11_ | 0.978 |
| 28 | Isorhamnetin-3-O-nehesperidine | 624.16887 | C_28_H_32_O_16_ | 6.853 |
| 29 | Nobiletin | 402.13151 | C_21_H_22_O_8_ | 10.796 |
| 30 | Baicalin | 446.08504 | C_21_H_18_O_11_ | 12.381 |
| 31 | Salicylic acid | 138.03164 | C_7_H_6_O_3_ | 9.672 |
| 32 | Acetophenone | 120.05764 | C_8_H_8_O | 4.781 |
| 33 | Hyperoside | 464.09556 | C_21_H_20_O_12_ | 4.992 |
| 34 | L-Glutamic acid | 147.05338 | C_5_H_9_NO_4_ | 1.627 |
| 35 | Ginkgolic Acid C15:1 | 346.25081 | C_22_H_34_O_3_ | 16.452 |
| 36 | Apocynin | 166.06326 | C_9_H_10_O_3_ | 5.385 |
| 37 | (+)-Magnoflorine | 341.16286 | C_20_H_23_NO_4_ | 4.574 |
| 38 | Indole-3-acrylic acid | 187.06351 | C_11_H_9_NO_2_ | 4.029 |
| 39 | Manninotriose | 504.16899 | C_18_H_32_O_16_ | 0.94 |
| 40 | 3-Methoxybenzaldehyde | 136.05264 | C_8_H_8_O_2_ | 4.145 |
| 41 | Tangeretin | 372.12105 | C_20_H_20_O_7_ | 11.385 |
| 42 | Epicatechin | 290.07915 | C_15_H_14_O_6_ | 3.46 |
| 43 | Procyanidin B2 | 578.14262 | C_30_H_26_O_12_ | 5.393 |
| 44 | Nicotinamide | 122.04824 | C_6_H_6_N_2_O | 2.045 |
| 45 | Nuciferine | 295.15737 | C_19_H_21_NO_2_ | 6.185 |
| 46 | Coumarin | 146.03688 | C_9_H_6_O_2_ | 9.256 |
| 47 | Cytosine | 111.04344 | C_4_H_5_N_3_O | 0.894 |
| 48 | Guanine | 151.04967 | C_5_H_5_N_5_O | 1.914 |
| 49 | Rosmarinic acid | 360.08466 | C_18_H_16_O_8_ | 7.979 |
| 50 | Citric acid | 192.02701 | C_6_H_8_O_7_ | 5.415 |
| 51 | D-Raffinose | 504.16915 | C_18_H_32_O_16_ | 0.952 |
| 52 | Wogonoside | 460.10081 | C_22_H_20_O_11_ | 11.255 |
| 53 | Wogonoside | 460.10039 | C_22_H_20_O_11_ | 13.332 |
| 54 | Kaempferol | 286.04775 | C_15_H_10_O_6_ | 12.39 |
| 55 | DL-Stachydrine | 143.0949 | C_7_H_13_NO_2_ | 0.912 |
| 56 | Naringin | 580.17949 | C_27_H_32_O_14_ | 5.583 |
| 57 | Nicotinic acid | 123.03228 | C_6_H_5_NO_2_ | 2.304 |
| 58 | 4-Hydroxybenzoic acid | 138.03164 | C_7_H_6_O_3_ | 3.649 |
| 59 | Amygdalin | 457.15866 | C_20_H_27_NO_11_ | 5.491 |
| 60 | Guanine | 151.04967 | C_5_H_5_N_5_O | 0.938 |
| 61 | Astragalin | 448.10061 | C_21_H_20_O_11_ | 6.858 |
| 62 | Isorhamnetin | 316.05834 | C_16_H_12_O_7_ | 6.837 |
| 63 | Epiberberine | 335.1158 | C_20_H_17_NO_4_ | 5.69 |
| 64 | Benzoic acid | 122.03691 | C_7_H_6_O_2_ | 4.174 |
| 65 | Calceolarioside B | 478.14744 | C_23_H_26_O_11_ | 5.201 |
| 66 | Cryptotanshinone | 296.14115 | C_19_H_20_O_3_ | 12.592 |
| 67 | 12-Oxophytodienoic acid | 292.20393 | C_18_H_28_O_3_ | 12.617 |
| 68 | L-Tryptophan | 204.09006 | C_11_H_12_N_2_O_2_ | 3.945 |
| 69 | 5-Hydroxymethylfurfural | 126.03185 | C_6_H_6_O_3_ | 3.267 |
| 70 | Shikimic acid | 174.05277 | C_7_H_10_O_5_ | 5.021 |
| 71 | Kaempferol | 286.04778 | C_15_H_10_O_6_ | 6.857 |
| 72 | Isoacteoside | 624.2053 | C_29_H_36_O_15_ | 5.101 |
| 73 | Kaempferol-3-O-rutinoside | 594.15843 | C_27_H_30_O_15_ | 6.783 |
| 74 | 7-Methoxycoumarin | 176.04755 | C_10_H_8_O_3_ | 8.774 |
| 75 | D-(-)-Glutamine | 146.06936 | C_5_H_10_N_2_O_3_ | 5.646 |
| 76 | Stachyose | 666.22152 | C_24_H_42_O_21_ | 0.944 |
| 77 | Oroxylin A-7-O-β-D-glucuronide | 460.10039 | C_22_H_20_O_11_ | 12.884 |
| 78 | Glycitein | 284.06865 | C_16_H_12_O_5_ | 10.757 |
| 79 | 2,5-Dimethylpyrazine | 108.06893 | C_6_H_8_N_2_ | 15.423 |
| 80 | Phenethylamine | 121.08934 | C_8_H_11_N | 1.288 |
| 81 | 4-Guanidinobutyric acid | 145.0853 | C_5_H_11_N_3_O_2_ | 0.807 |
| 82 | Lithospermic acid | 538.1114 | C_27_H_22_O_12_ | 10.97 |
| 83 | Asparagine | 132.05367 | C_4_H_8_N_2_O_3_ | 0.864 |
| 84 | Wogonin | 284.06856 | C_16_H_12_O_5_ | 9.224 |
| 85 | Lariciresinol 4-O-glucoside | 522.20842 | C_26_H_34_O_11_ | 6.458 |
| 86 | Caffeic acid | 180.04241 | C_9_H_8_O_4_ | 7.09 |
| 87 | Phloridzin | 436.1371 | C_21_H_24_O_10_ | 6.46 |
| 88 | Sinensetin | 372.12105 | C_20_H_20_O_7_ | 10.231 |
| 89 | Baohuoside I | 514.1841 | C_27_H_30_O_10_ | 11.066 |
| 90 | Dehydroevodiamine | 301.1216 | C_19_H_15_N_3O_ | 5.704 |
| 91 | D-(-)-Quinic acid | 192.06369 | C_7_H_12_O_6_ | 2.689 |
| 92 | 1-Naphthol | 144.05766 | C_10_H_8_O | 6.149 |
| 93 | Vanillin | 152.0473 | C_8_H_8_O_3_ | 4.822 |
| 94 | 10-HDA | 186.12582 | C_10_H_18_O_3_ | 6.378 |
| 95 | L-Aspartic acid | 133.03775 | C_4_H_7_NO_4_ | 2.268 |
| 96 | Calycosin | 284.06865 | C_16_H_12_O_5_ | 8.351 |
| 97 | 5,2'-Dihydroxy-6,7,8,6'-tetramethoxyflavone | 374.10036 | C_19_H_18_O_8_ | 10.914 |
| 98 | 2-Aminooctadec-4-yne-1,3-diol | 297.26685 | C_18_H_35_NO_2_ | 9.787 |
| 99 | Formononetin | 268.07372 | C_16_H_12_O_4_ | 9.92 |
| 100 | 9-Oxo-10(E),12(E)-octadecadienoic acid | 294.21959 | C_18_H_30_O_3_ | 12.971 |
| 101 | Asiatic acid | 488.35022 | C_30_H_48_O_5_ | 11.657 |
| 102 | Gentiopicrin | 356.11078 | C_16_H_20_O_9_ | 3.581 |
| 103 | L-Tyrosine | 181.07411 | C_9_H_11_NO_3_ | 0.931 |
| 104 | Cordycepin | 251.10199 | C_10_H_13_N_5_O_3_ | 3.78 |
| 105 | 1,3-Dicaffeoylquinic acid | 516.12683 | C_25_H_24_O_12_ | 7.809 |
| 106 | Isochlorogenic acid C | 516.12672 | C_25_H_24_O_12_ | 8.511 |
| 107 | Loganic acid | 376.13695 | C_16_H_24_O_10_ | 4.281 |
| 108 | 4-Methyl-6,7-dihydroxycoumarin | 192.0423 | C_10_H_8_O_4_ | 6.944 |
| 109 | Emodin-8-O-β-D-glucopyranoside | 432.10567 | C_21_H_20_O_10_ | 7.084 |
| 110 | 3-Hydroxypyridine | 95.03724 | C_5_H_5_NO | 1.187 |
| 111 | Puerarin | 416.11092 | C2_1_H_20_O_9_ | 5.544 |
| 112 | 4-Methoxycinnamaldehyde | 162.06817 | C_10_H_10_O_2_ | 4.997 |
| 113 | Hexadecanamide | 255.25629 | C_16_H_33_NO | 13.951 |
| 114 | Danshensu | 198.0525 | C_9_H_10_O_5_ | 7.732 |
| 115 | Eleutheroside B | 372.14182 | C_17_H_24_O_9_ | 5.236 |
| 116 | Grosvenorine | 740.21562 | C_33_H_40_O_19_ | 6.244 |
| 117 | Adenosine 3'5'-cyclic monophosphate | 329.05251 | C_10_H_12_N_5_O_6_P | 6.811 |
| 118 | Icaritin | 368.12605 | C_21_H_20_O_6_ | 11.066 |
| 119 | Naringenin | 272.0685 | C_15_H_12_O_5_ | 7.103 |
| 120 | Icariin | 676.23668 | C_33_H_40_O_15_ | 8.588 |
| 121 | Purpureaside C | 786.25783 | C_35_H_46_O_20_ | 5.892 |
| 122 | Glabrolide | 468.32427 | C_30_H_44_O_4_ | 11.868 |
| 123 | Dihydrotanshinone I | 278.09443 | C_18_H_14_O_3_ | 11.946 |
| 124 | Hispidulin | 300.06331 | C_16_H_12_O_6_ | 7.498 |
| 125 | Oleuropein | 540.18469 | C_25_H_32_O_13_ | 7.675 |
| 126 | Styraxlignolide F | 534.20948 | C_27_H_34_O_11_ | 8.176 |
| 127 | Oleamide | 281.27187 | C_18_H_35_NO | 14.109 |
| 128 | Hesperetin | 302.07929 | C_16_H_14_O_6_ | 7.644 |
| 129 | Glaucine | 355.17839 | C_21_H_25_NO_4_ | 5.864 |
| 130 | 18 β-Glycyrrhetintic Acid | 470.33968 | C_30_H_46_O_4_ | 12.713 |
| 131 | 6-Methylquinoline | 143.07373 | C_10_H_9_N | 5.127 |
| 132 | Gentisic acid | 154.02662 | C_7_H_6_O_4_ | 4.013 |
| 133 | Oroxylin A | 284.06865 | C_16_H_12_O_5_ | 11.04 |
| 134 | Tetrahydropalmatine | 355.17824 | C_21_H_25_NO_4_ | 6.235 |
| 135 | L-Glutamine | 146.06927 | C_5_H_10_N_2_O_3_ | 6.839 |
| 136 | Perillene | 150.1047 | C_10_H_14_O | 7.29 |
| 137 | Luteolin | 286.0479 | C_15_H_10_O_6_ | 6.783 |
| 138 | Tanshinone IIA | 294.12567 | C_19_H_18_O_3_ | 13.085 |
| 139 | Hydroprotopine | 353.12654 | C_20_H_19_NO_5_ | 5.336 |
| 140 | Quillaic acid | 486.33464 | C_30_H_46_O_5_ | 10.431 |
| 141 | Bis(4-ethylbenzylidene) sorbitol | 414.20425 | C_24_H_30_O_6_ | 11.807 |
| 142 | Salvianolic acid C | 492.10579 | C_26_H_20_O_10_ | 9.431 |
| 143 | Clareolide | 250.19328 | C_16_H_26_O_2_ | 10.846 |
| 144 | Peiminine | 429.32454 | C_27_H_43_NO_3_ | 5.836 |
| 145 | Salvianolic acid A | 494.12099 | C_26_H_22_O_10_ | 15.82 |
| 146 | Vicenin II | 594.15868 | C_27_H_30_O_15_ | 5.618 |
| 147 | p-Coumaric acid | 164.04759 | C_9_H_8_O_3_ | 1.292 |
| 148 | Neohesperidin | 610.18983 | C_28_H_34_O_15_ | 5.713 |
| 149 | 3,4,5-Trimethoxyphenyl 6-O-pentopyranosyl-β-D-glucopyranoside | 478.16859 | C_20_H_30_O_13_ | 5.451 |
| 150 | Ononin | 430.1267 | C_22_H_22_O_9_ | 7.907 |
| 151 | 7,8-Dihydroxycoumarin | 178.02659 | C_9_H_6_O_4_ | 7.735 |
| 152 | Curcumenol | 234.16217 | C_15_H_22_O_2_ | 11.33 |
| 153 | 4-Indolecarbaldehyde | 145.05297 | C_9_H_7_NO | 4.022 |
| 154 | 6-Gingerol | 294.18334 | C_17_H_26_O_4_ | 10.105 |
| 155 | Casticin | 374.10029 | C_19_H_18_O_8_ | 9.4 |
| 156 | Rosamultin | 650.40257 | C_36_H_58_O_10_ | 10.04 |
| 157 | Ferulaldehyde | 178.06319 | C_10_H_10_O_3_ | 7.389 |
| 158 | 3,4-Dihydroxybenzaldehyde | 138.03186 | C_7_H_6_O_3_ | 5.645 |
| 159 | Daidzein | 254.05795 | C_15_H_10_O_4_ | 8.008 |
| 160 | Eriodictyol | 288.0635 | C_15_H_12_O_6_ | 6.474 |
| 161 | Abscisic acid | 264.13624 | C_15_H_20_O_4_ | 6.981 |
| 162 | 4-Methoxyphenylacetic acid | 166.0628 | C_9_H_10_O_3_ | 3.823 |
| 163 | Docosanamide | 339.35021 | C_22_H_45_NO | 14.598 |
| 164 | Daidzin | 416.11058 | C_21_H_20_O_9_ | 6.075 |
| 165 | α-Linolenic acid | 278.22454 | C_18_H_30_O_2_ | 9.679 |
| 166 | Uridine | 244.06946 | C_9_H_12_N_2_O_6_ | 1.067 |
| 167 | Linoleoyl ethanolamide | 323.28237 | C_20_H_37_NO_2_ | 13.326 |
| 168 | 5-O-Demethylnobiletin | 388.11574 | C_20_H_20_O_8_ | 11.652 |
| 169 | Tectoridin | 462.11641 | C_22_H_22_O_11_ | 7.305 |
| 170 | Kurarinone | 438.20418 | C_26_H_30_O_6_ | 11.22 |
| 171 | Poricoic acid A | 498.33446 | C_31_H_46_O_5_ | 12.531 |
| 172 | Cynaroside | 448.10063 | C_21_H_20_O_11_ | 5.148 |
| 173 | Crotonic acid | 86.03688 | C_4_H_6_O_2_ | 9.684 |
| 174 | Citropten | 206.05818 | C_11_H_10_O_4_ | 7.754 |
| 175 | Liquiritigenin | 256.07359 | C_15_H_12_O_4_ | 6.634 |
| 176 | Germacrone | 218.16704 | C_15_H_22_O | 12.595 |
| 177 | Epigallocatechin | 306.07402 | C_15_H_14_O_7_ | 3.462 |
| 178 | Medicarpin | 270.08937 | C_16_H_14_O_4_ | 9.148 |
| 179 | Secoxyloganin | 404.13154 | C_17_H_24_O_11_ | 5.426 |
| 180 | Procyanidin A2 | 576.12683 | C_30_H_24_O_12_ | 5.398 |
| 181 | Quercetin 3-O-β-D-Glucuronide | 478.07487 | C_21_H_18_O_13_ | 8.491 |
| 182 | Isoliquiritin | 418.12654 | C_21_H_22_O_9_ | 6.245 |
| 183 | Rhoifolin | 578.16283 | C_27_H_30_O_14_ | 6.94 |
| 184 | Sinapic acid | 224.06837 | C_11_H_12_O_5_ | 5.375 |
| 185 | Calycosin-7-O-β-D-glucoside | 446.12135 | C_22_H_22_O_10_ | 6.508 |
| 186 | Apigenin | 270.05293 | C_15_H_10_O_5_ | 6.711 |
| 187 | Pinoresinol 4-O-glucoside | 520.19447 | C_26_H_32_O_11_ | 5.493 |
| 188 | Lupenone | 424.37073 | C_30_H_48_O | 11.747 |
| 189 | Skimmin | 324.08458 | C_15_H_16_O_8_ | 7.152 |
| 190 | Allocryptopine | 369.1577 | C_21_H_23_NO_5_ | 5.584 |
| 191 | Hydroxygenkwanin | 300.0634 | C_16_H_12_O_6_ | 9.156 |
| 192 | Diosmetin | 300.0634 | C_16_H_12_O_6_ | 9.031 |
| 193 | 3,5-Dimethoxy-4-hydroxybenzaldehyde | 182.058 | C_9_H_10_O_4_ | 5.157 |
| 194 | Liquiritin | 418.12575 | C_21_H_22_O_9_ | 5.102 |
| 195 | Norisoboldine | 313.13103 | C_18_H_19_NO_4_ | 4.316 |
| 196 | Jaceosidin | 330.0739 | C_17_H_14_O_7_ | 9.687 |
| 197 | Pyridoxine | 169.07414 | C_8_H_1_1NO_3_ | 1.186 |
| 198 | Honokiol | 266.13072 | C_18_H_18_O_2_ | 11.021 |
| 199 | Hesperidin | 610.18968 | C_28_H_34_O_15_ | 7.23 |
| 200 | Cinnamaldehyde | 132.05768 | C_9_H_8_O | 7.286 |
| 201 | Wilforlide A | 454.34441 | C_30_H_46_O_3_ | 10.788 |
| 202 | Camphor | 152.12027 | C_10_H_16_O | 9.744 |
| 203 | Cimifugin | 306.11034 | C_16_H_18_O_6_ | 6.854 |
| 204 | Procyanidin B1 | 578.14263 | C_30_H_26_O_12_ | 6.484 |
| 205 | Vicenin III | 564.14778 | C_26_H_28_O_14_ | 4.143 |
| 206 | Scutellarein | 286.0479 | C_15_H_10_O_6_ | 6.243 |
| 207 | Genistein | 270.0529 | C_15_H_10_O_5_ | 5.802 |
| 208 | Cyclo(leucylprolyl) | 210.13703 | C_11_H_18_N_2_O_2_ | 6.435 |
| 209 | Sinomenine | 329.16271 | C_19_H_23_NO_4_ | 4.625 |
| 210 | 4'-O-Glucosylvitexin | 594.15868 | C_27_H_30_O_15_ | 5.923 |
| 211 | Isovitexin | 432.10573 | C_21_H_20_O_10_ | 6.481 |
| 212 | Scutellarin | 462.07997 | C_21_H_18_O_12_ | 11.922 |
| 213 | Sophoricoside | 432.10573 | C_21_H_20_O_10_ | 6.716 |
| 214 | 1-Linoleoyl glycerol | 354.27696 | C_21_H_38_O_4_ | 12.976 |
| 215 | Senkyunolide A | 192.11523 | C_12_H_16_O_2_ | 8.16 |
| 216 | Poncirin | 594.19457 | C_28_H_34_O_14_ | 8.478 |
| 217 | Chrysin | 254.05809 | C_15_H_10_O_4_ | 10.85 |
| 218 | Quercetin | 302.04265 | C_15_H_10_O_7_ | 15.781 |
| 219 | Neobavaisoflavone | 322.12041 | C_20_H_18_O_4_ | 11.13 |
| 220 | 4-Ethylbenzaldehyde | 134.07334 | C_9_H_10_O | 6.907 |
| 221 | Glycitin | 446.12148 | C_22_H_22_O_10_ | 8.578 |
| 222 | Formononetin | 268.07359 | C_16_H_12_O_4_ | 10.664 |
| 223 | Eupafolin | 316.05849 | C_16_H_12_O_7_ | 7.604 |
| 224 | Evodiamine | 303.13738 | C_19_H_17_N_3_O | 11.257 |
| 225 | Icarisid I | 530.17894 | C_27_H_30_O_11_ | 8.594 |
| 226 | Caffeine | 194.08054 | C_8_H_10_N_4_O_2_ | 5.31 |
| 227 | L- (+)-Arginine | 174.11189 | C_6_H_14_N_4_O_2_ | 15.801 |
| 228 | 3,5-di-tert-Butyl-4-hydroxybenzaldehyde | 234.16221 | C_15_H_22_O_2_ | 12.628 |
| 229 | Epmedin B | 808.27867 | C_38_H_48_O_19_ | 8.34 |
| 230 | Diphenylamine | 169.08928 | C_12_H_11_N | 12.039 |
| 231 | Curcumin | 368.12593 | C_21_H_20_O_6_ | 9.677 |
| 232 | Forsythoside I | 624.20543 | C_29_H_36_O_15_ | 6.615 |

**Table S2 The 34 components with OB value ≥ 30% and DL value ≥ 0.18 of TCMSP**

| No. | Compound | OB | DL | | Structural classification |
| --- | --- | --- | --- | --- | --- |
| 1 | Berberine | 36.86% | 0.78 | Alkaloids | |
| 2 | Morin | 46.23% | 0.27 | Flavonoids | |
| 3 | Nobiletin | 61.67% | 0.52 | Flavonoids | |
| 4 | Nuciferine | 34.43% | 0.40 | Alkaloids | |
| 5 | Isorhamnetin | 49.60% | 0.31 | Flavonoids | |
| 6 | Epiberberine | 30.74% | 0.55 | Alkaloids | |
| 7 | Cryptotanshinone | 52.34% | 0.40 | Quinones | |
| 8 | Kaempferol | 41.88% | 0.24 | Flavonoids | |
| 9 | Wogonin | 30.68% | 0.23 | Flavonoids | |
| 10 | Sinensetin | 50.56% | 0.45 | Flavonoids | |
| 11 | Calycosin | 47.75% | 0.24 | Flavonoids | |
| 12 | Formononetin | 69.67% | 0.21 | Flavonoids | |
| 13 | Icariin | 41.58% | 0.61 | Flavonoids | |
| 14 | Hispidulin | 30.97% | 0.27 | Flavonoids | |
| 15 | Hesperetin | 70.31% | 0.27 | Flavonoids | |
| 16 | Oroxylin A | 41.37% | 0.23 | Flavonoids | |
| 17 | Tetrahydropalmatine | 73.94% | 0.64 | Alkaloids | |
| 18 | Luteolin | 36.16% | 0.25 | Flavonoids | |
| 19 | Tanshinone IIA | 49.89% | 0.40 | Terpenoids | |
| 20 | Neohesperidin | 57.44% | 0.27 | Flavonoids | |
| 21 | Eriodictyol | 71.79% | 0.24 | Flavonoids | |
| 22 | Poricoic acid A | 30.61% | 0.76 | Terpenoids | |
| 23 | Liquiritigenin | 32.76% | 0.18 | Flavonoids | |
| 24 | Medicarpin | 49.22% | 0.34 | Flavonoids | |
| 25 | 1,3-Dicaffeoylquinic acid | 31.76% | 0.68 | Phenylpropanoids | |
| 26 | Skimmin | 38.35% | 0.32 | Phenylpropanoids | |
| 27 | Hydroxygenkwanin | 36.47% | 0.27 | Flavonoids | |
| 28 | Procyanidin B1 | 67.87% | 0.66 | Flavonoids | |
| 29 | Sinomenine | 30.98% | 0.46 | Alkaloids | |
| 30 | Isovitexin | 31.29% | 0.72 | Flavonoids | |
| 31 | Quercetin | 46.43% | 0.28 | Flavonoids | |
| 32 | Poncirin | 36.55% | 0.74 | Flavonoids | |
| 33 | Glycitin | 50.48% | 0.24 | Flavonoids | |
| 34 | Evodiamine | 86.02% | 0.64 | Alkaloids | |

**Table S3 Abbreviations for all cancers**

| Abbreviation | English name |
| --- | --- |
| ACC | Adrenocortical carcinoma |
| BLCA | Bladder Urothelial Carcinoma |
| BRCA | Breast invasive carcinoma |
| CESC | Cervical squamous cell carcinoma and endocervical adenocarcinoma |
| CHOL | Cholangiocarcinoma |
| COAD | Colon adenocarcinoma |
| DLBC | Lymphoid Neoplasm Diffuse Large B-cell Lymphoma |
| ESCA | Esophageal carcinoma |
| GBM | Glioblastoma multiforme |
| HNSC | Head and Neck squamous cell carcinoma |
| KICH | Kidney Chromophobe |
| KIRC | Kidney renal clear cell carcinoma |
| KIRP | Kidney renal papillary cell carcinoma |
| LAML | Acute Myeloid Leukemia |
| LGG | Brain Lower Grade Glioma |
| LIHC | Liver hepatocellular carcinoma |
| LUAD | Lung adenocarcinoma |
| LUSC | Lung squamous cell carcinoma |
| MESO | Mesothelioma |
| OV | Ovarian serous cystadenocarcinoma |
| PAAD | Pancreatic adenocarcinoma |
| PCPG | Pheochromocytoma and Paraganglioma |
| PRAD | Prostate adenocarcinoma |
| READ | Rectum adenocarcinoma |
| SARC | Sarcoma |
| STAD | Stomach adenocarcinoma |
| SKCM | Skin Cutaneous Melanoma |
| TGCT | Testicular Germ Cell Tumors |
| THCA | Thyroid carcinoma |
| THYM | Thymoma |
| UCEC | Uterine Corpus Endometrial Carcinoma |
| UCS | Uterine Carcinosarcoma |
| UVM | Uveal Melanoma |

**Supplementary figures and legends**

**Figure S1.** **HPLC superposed fingerprint of 9 batches of SQWCF granules. (A)** HPLC control(S1) fingerprint. **(B)** HPLC superposed fingerprint.

**Figure S2. Molecular docking results. (A)** CASP3 and core compounds of SQWCF. **(B)** Bcl-2 and core compounds of SQWCF. **(C)** FAM81A and core compounds of SQWCF. All pictures show the 3D docking of ligands in the active binding pocket (left), with the hydrophobic effect area and the 2D interaction patterns between the ligands and proteins (right).

**Figure S3. Expression of PCNT and TMX4 in pan-cancer and their prognosis in gastric cancer. (A)** PCNT expression in TCGA tumors and normal tissues with the data of the GTEx database as controls. **(B)** PCNT expression in TCGA tumors and adjacent normal tissues. **(C)** The Kaplan–Meier survival curves of high and low PCNT expression in GC through the Kaplan–Meier plotter database. **(D)** TMX4 expression in TCGA tumors and normal tissues with the data of the GTEx database as controls. **(E)** TMX4 expression in TCGA tumors and adjacent normal tissues. **(F)** The Kaplan–Meier survival curves of high and low TMX4 expression in GC through the Kaplan–Meier plotter database. *p < 0.05, **p < 0.01, ***p < 0.001, NS, no significant.
